# Dapagliflozin across the range of ejection fraction in patients with heart failure: a patient-level, pooled meta-analysis of DAPA-HF and DELIVER

**DOI:** 10.1038/s41591-022-01971-4

**Published:** 2022-08-27

**Authors:** Pardeep S. Jhund, Toru Kondo, Jawad H. Butt, Kieran F. Docherty, Brian L. Claggett, Akshay S. Desai, Muthiah Vaduganathan, Samvel B. Gasparyan, Olof Bengtsson, Daniel Lindholm, Magnus Petersson, Anna Maria Langkilde, Rudolf A. de Boer, David DeMets, Adrian F. Hernandez, Silvio E. Inzucchi, Mikhail N. Kosiborod, Lars Køber, Carolyn S. P. Lam, Felipe A. Martinez, Marc S. Sabatine, Sanjiv J. Shah, Scott D. Solomon, John J. V. McMurray

**Affiliations:** 1 BHF Glasgow Cardiovascular Research Centre, School of Cardiovascular and Metabolic Health, University of Glasgow, Glasgow, UK; 2 Cardiovascular Division, Brigham and Women’s Hospital, Harvard Medical School, Boston, MA USA; 3 Late-Stage Development, Cardiovascular, Renal, and Metabolism, BioPharmaceuticals R&D, AstraZeneca, Gothenburg, Sweden; 4 Department of Cardiology, University Medical Center Groningen, University of Groningen, Groningen, the Netherlands; 5 University of Wisconsin, Madison, WI USA; 6 Duke University Medical Center, Durham, NC USA; 7 Yale School of Medicine, New Haven, CT USA; 8 Saint Luke’s Mid America Heart Institute, University of Missouri-Kansas City, Kansas City, MO USA; 9 Department of Cardiology, Copenhagen University Hospital–Rigshospitalet, Copenhagen, Denmark; 10 National Heart Centre Singapore & Duke-National University of Singapore, Singapore, Singapore; 11 University of Cordoba, Cordoba, Argentina; 12 TIMI Study Group, Division of Cardiovascular Medicine, Brigham and Women’s Hospital, Boston, MA USA; 13 Northwestern University Feinberg School of Medicine, Chicago, IL USA

**Keywords:** Diseases, Heart failure

## Abstract

Whether the sodium–glucose cotransporter 2 inhibitor dapagliflozin reduces the risk of a range of morbidity and mortality outcomes in patients with heart failure regardless of ejection fraction is unknown. A patient-level pooled meta-analysis of two trials testing dapagliflozin in participants with heart failure and different ranges of left ventricular ejection fraction (≤40% and >40%) was pre-specified to examine the effect of treatment on endpoints that neither trial, individually, was powered for and to test the consistency of the effect of dapagliflozin across the range of ejection fractions. The pre-specified endpoints were: death from cardiovascular causes; death from any cause; total hospital admissions for heart failure; and the composite of death from cardiovascular causes, myocardial infarction or stroke (major adverse cardiovascular events (MACEs)). A total of 11,007 participants with a mean ejection fraction of 44% (s.d. 14%) were included. Dapagliflozin reduced the risk of death from cardiovascular causes (hazard ratio (HR) 0.86, 95% confidence interval (CI) 0.76–0.97; *P* = 0.01), death from any cause (HR 0.90, 95% CI 0.82–0.99; *P* = 0.03), total hospital admissions for heart failure (rate ratio 0.71, 95% CI 0.65–0.78; *P* < 0.001) and MACEs (HR 0.90, 95% CI 0.81–1.00; *P* = 0.045). There was no evidence that the effect of dapagliflozin differed by ejection fraction. In a patient-level pooled meta-analysis covering the full range of ejection fractions in patients with heart failure, dapagliflozin reduced the risk of death from cardiovascular causes and hospital admissions for heart failure (PROSPERO: CRD42022346524).

## Abstract (web summary)

A pre-specified meta-analysis of pooled, individual patient-level data from the DELIVER and DAPA-HF trials, testing dapagliflozin in patients with heart failure, demonstrates reductions in risk of cardiovascular-associated deaths and hospital admissions for heart failure, regardless of ejection fraction.

## Main

Sodium–glucose cotransporter 2 (SGLT2) inhibitors have been shown to be of benefit in patients with heart failure (HF), leading to significant reductions in the composite outcome of worsening HF (often leading to hospitalization) or death from cardiovascular (CV) causes^1–5^. We planned a prospective, patient-level pooled meta-analysis of the Dapagliflozin and Prevention of Adverse Outcomes in Heart Failure (DAPA-HF) and Dapagliflozin Evaluation to Improve the LIVEs of Patients With Preserved Ejection Fraction Heart Failure (DELIVER) trials to provide additional data about the efficacy and safety of dapagliflozin as a treatment for patients with HF^1,2^. The individual trials were powered for their primary composite endpoints^6,7^ and the purpose of the pooled analysis was to evaluate the key components of these endpoints and important secondary efficacy outcomes that required more power than provided by the individual trials. In particular, we pre-specified examination of the effect of dapagliflozin on mortality and the composite of death from CV causes, myocardial infarction (MI) or stroke (MACE). We also pre-specified that these outcomes would be examined in a limited number of patient subgroups to examine the consistency of the effects of dapagliflozin. One of these, left ventricular ejection fraction (LVEF), has become a key clinical question since the pooled analysis was originally conceived^8^. Treatments for heart failure that work through neurohumoral pathways have their greatest benefit in patients with a reduced LVEF, that is, ≤40%. Analyses of trials testing such treatments demonstrated attenuated benefit in patients with an ejection fraction >55–60%^9–11^. This pattern is considered biologically plausible because patients with lower ejection fractions exhibit greater neurohumoral activation than patients with higher ejection fractions^9–11^. SGLT2 inhibitors are not thought to act through neurohumoral pathways and no gradient in their effect related to ejection fraction was anticipated. However, a pooled analysis of the EMPagliflozin outcomE tRial in patients with chrOnic heaRt failure (EMPEROR) trials unexpectedly suggested a similar pattern of attenuated benefit in patients with a normal ejection fraction^3,4,12^. If correct, this finding has major implications for the treatment of patients with HF, a large proportion of whom have a normal ejection fraction, as well as our understanding of the pathophysiology of this syndrome and how SGLT2 inhibitors exert their benefits in HF. For this reason, before DELIVER^2^ was unblinded, we prepared an updated statistical analysis plan to pre-specify additional analyses of the effects of dapagliflozin across the full range of LVEF at baseline (Supplementary information).

## Results

### Patient-level pooled meta-analysis of DAPA-HF and DELIVER

**Extended Data Fig. 1:**
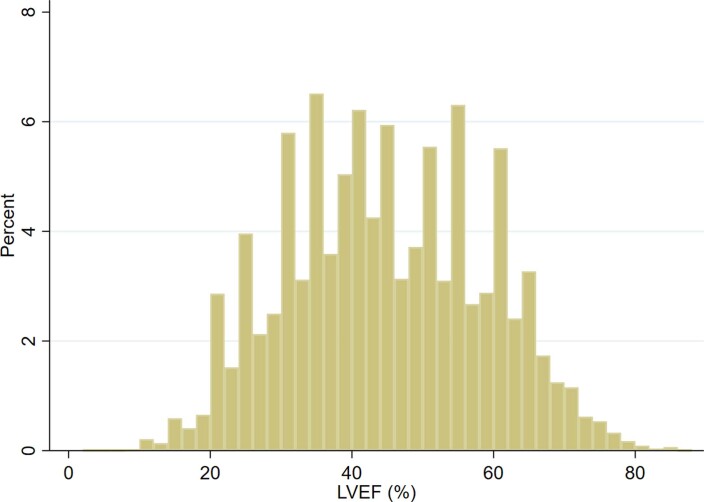
Distribution of LVEF in pooled DAPA-HF and DELIVER dataset complete. Distribution of left ventricular ejection fraction (LVEF) in the total population in DAPA-HF and DELIVER.

Of the 11,007 participants included in this analysis, 4,744 had an LVEF ≤ 40% and 6,263 an ejection fraction >40%, with 5,503 randomized to placebo and 5,504 randomized to dapagliflozin. The distributions of LVEFs in the overall population are shown in Extended Data Fig. 1. The mean LVEF was 44% (s.d. 14%) and the median 44% (interquartile range (IQR) 34–55%). The median follow-up was 22 months (IQR 17–30 months).

### Baseline characteristics

Compared with participants with a lower ejection fraction, those with a higher ejection fraction were older and more likely to be a woman (Table 1). Blood pressure was 11 mmHg higher and body mass index (BMI) was 2 kg m^−2^ higher in those with an ejection fraction >60% compared with ≤30%. A history of hypertension and atrial fibrillation was more common and that of MI less common in patients with higher ejection fractions. The proportion of patients in New York Heart Association (NYHA) class III/IV was lower among those with a higher ejection fraction but patient-reported health status, measured by the Kansas City Cardiomyopathy Questionnaire—Total symptom score (KCCQ-TSS), was worse in participants with higher ejection fractions. Both N-terminal pro-brain natriuretic peptide (NT-proBNP) and estimated glomerular filtration rate (eGFR) were lower in the patients with higher ejection fraction, as was the use of angiotensin-converting enzyme (ACE) inhibitors, angiotensin receptor blockers (ARBs), sacubitril/valsartan, β-blockers, mineralocorticoid receptor antagonists (MRAs) and intracardiac devices.

**Table 1:** Baseline characteristics of the pooled DAPA-HF and DELIVER cohort by ejection fraction category

	≤30%	>30 and ≤37%	>37 and ≤44%	>44 and ≤51%	>51 and ≤60%	>60%	*P* for trend
	*N* = 2,161	*N* = 1,584	*N* = 1,863	*N* = 1,862	*N* = 2,142	*N* = 1,395	
LVEF (%)	24.9 ± 4.7	34.4 ± 1.8	40.6 ± 1.9	47.7 ± 2.2	56.4 ± 2.7	66.6 ± 4.6	
Randomized treatment: no. (%)							0.27
Placebo	1,099 (50.9)	785 (49.6)	900 (48.3)	947 (50.9)	1,054 (49.2)	718 (51.5)	
Dapagliflozin	1,062 (49.1)	799 (50.4)	963 (51.7)	915 (49.1)	1,088 (50.8)	677 (48.5)	
Age (years)	65 ± 11	67 ± 11	69 ± 10	70 ± 10	73 ± 9	74 ± 9	<0.001
Sex: no. (%)							<0.001
Female	445 (20.6)	379 (23.9)	528 (28.3)	667 (35.8)	1,053 (49.2)	784 (56.2)	
Male	1,716 (79.4)	1,205 (76.1)	1,335 (71.7)	1,195 (64.2)	1,089 (50.8)	611 (43.8)	
Region: no. (%)							<0.001
Europe and Saudi Arabia	804 (37.2)	757 (47.8)	1,017 (54.6)	1,060 (56.9)	1,075 (50.2)	446 (32.0)	
North America	381 (17.6)	195 (12.3)	162 (8.7)	210 (11.3)	360 (16.8)	220 (15.8)	
South America	431 (19.9)	271 (17.1)	315 (16.9)	310 (16.6)	318 (14.8)	353 (25.3)	
Asia/Pacific	545 (25.2)	361 (22.8)	369 (19.8)	282 (15.1)	389 (18.2)	376 (27.0)	
Race: no. (%)							<0.001
White	1,423 (65.8)	1,133 (71.5)	1,387 (74.4)	1,442 (77.4)	1,554 (72.5)	833 (59.7)	
Asian	554 (25.6)	367 (23.2)	379 (20.3)	293 (15.7)	404 (18.9)	393 (28.2)	
Black or African–American	147 (6.8)	59 (3.7)	33 (1.8)	42 (2.3)	59 (2.8)	45 (3.2)	
Other	37 (1.7)	25 (1.6)	64 (3.4)	85 (4.6)	125 (5.8)	124 (8.9)	
Pulse (beats min^−1^)	72 ± 12	71 ± 12	71 ± 11	72 ± 12	72 ± 12	71 ± 12	0.047
Systolic blood pressure (mmHg)	118 ± 15	124 ± 17	126 ± 15	128 ± 15	129 ± 15	129 ± 15	<0.001
Diastolic blood pressure (mmHg)	72 ± 10	74 ± 11	75 ± 10	75 ± 10	74 ± 11	73 ± 10	0.002
BMI (kg m^−2^)	28 ± 6	28 ± 6	29 ± 6	30 ± 6	30 ± 6	30 ± 6	<0.001
**Clinical history**							
Hypertension: no. (%)	1,463 (67.7)	1,221 (77.1)	1,565 (84.0)	1,646 (88.4)	1,937 (90.4)	1,244 (89.2)	<0.001
Type 2 diabetes mellitus: no. (%)	885 (41.0)	661 (41.7)	838 (45.0)	844 (45.3)	952 (44.4)	609 (43.7)	0.16
Stroke: no. (%)	207 (9.6)	149 (9.4)	184 (9.9)	166 (8.9)	236 (11.0)	121 (8.7)	0.19
MI: no. (%)	940 (43.5)	704 (44.4)	799 (42.9)	635 (34.1)	449 (21.0)	204 (14.6)	<0.001
Atrial fibrillation: no. (%)	736 (34.1)	635 (40.1)	811 (43.5)	1,014 (54.5)	1,291 (60.3)	796 (57.1)	<0.001
HF hospitalization: no. (%)	1,063 (49.2)	735 (46.4)	860 (46.2)	835 (44.8)	843 (39.4)	454 (32.5)	<0.001
NYHA II or III/IV: no. (%)							<0.001
II	1,466 (67.8)	1,065 (67.2)	1,277 (68.5)	1,369 (73.5)	1,641 (76.6)	1,098 (78.8)	
III/IV	695 (32.2)	519 (32.8)	586 (31.5)	493 (26.5)	501 (23.4)	296 (21.2)	
KCCQ-TSS	78 (59–93)	78 (59–92)	75 (57–91)	74 (56–90)	71 (54–86)	73 (54–88)	<0.001
NT-proBNP (ng l^−1^)	1680 (964–3163)	1309 (805–2362)	1225 (714–2225)	1089 (653–1877)	976 (632–1631)	903 (542–1548)	<0.001
eGFR (ml per min per 1.73 m^2^)	66 ± 20	66 ± 20	64 ± 19	62 ± 19	60 ± 18	59 ± 19	<0.001
Creatinine (µmol l^−1^)	106 ± 31	104 ± 30	103 ± 30	103 ± 31	102 ± 31	101 ± 32	<0.001
**Baseline treatment: no. (%)**							
Diuretics	1,876 (86.8)	1,312 (82.8)	1,565 (84.0)	1,645 (88.3)	1,952 (91.1)	1,238 (88.7)	<0.001
ACEi or ARB	1,714 (79.3)	1,339 (84.5)	1,516 (81.4)	1,381 (74.2)	1,549 (72.3)	996 (71.4)	<0.001
ARNI	306 (14.2)	153 (9.7)	162 (8.7)	107 (5.7)	60 (2.8)	21 (1.5)	<0.001
ACEi, ARB or ARNI	2,009 (93.0)	1,488 (93.9)	1,671 (89.7)	1,483 (79.6)	1,606 (75.0)	1,017 (72.9)	<0.001
β-Blocker	2,079 (96.2)	1,529 (96.5)	1,689 (90.7)	1,617 (86.8)	1,741 (81.3)	1,080 (77.4)	<0.001
MRA	1,610 (74.5)	1,124 (71.0)	1,149 (61.7)	853 (45.8)	821 (38.3)	480 (34.4)	<0.001
Digitalis	472 (21.8)	273 (17.2)	185 (9.9)	89 (4.8)	106 (4.9)	58 (4.2)	<0.001
CRT-D or CRT-P	202 (9.3)	104 (6.6)	68 (3.7)	43 (2.3)	31 (1.4)	6 (0.4)	0.002
CRT-D or ICD	772 (35.7)	329 (20.8)	187 (10.0)	74 (4.0)	39 (1.8)	9 (0.6)	<0.001

ACEi, ACE inhibitor; ARB, angiotensin receptor blocker; ARNI, angiotensin receptor neprilysin inhibitor; BMI, body mass index; CRT-D, cardiac resynchronization therapy—defibrillator; CRT-P, cardiac resynchronization therapy—pacemaker; eGFR, estimated glomerular filtration rate; HF, heart failure; ICD, implantable cardioverter defibrillator; KCCQ-TSS, Kansas City Cardiomyopathy Questionnaire Total Symptom Score; MI, myocardial infarction; MRA, mineralocorticoid receptor antagonist; NT-proBNP, N-terminal pro-B-type natriuretic peptide; NYHA, New York Heart Association. *P* values are two sided and calculated from Cochrane, Armitage and Cuzick’s tests across quantiles.

### Effect of dapagliflozin on outcomes according to ejection fraction

**Fig. 1:**
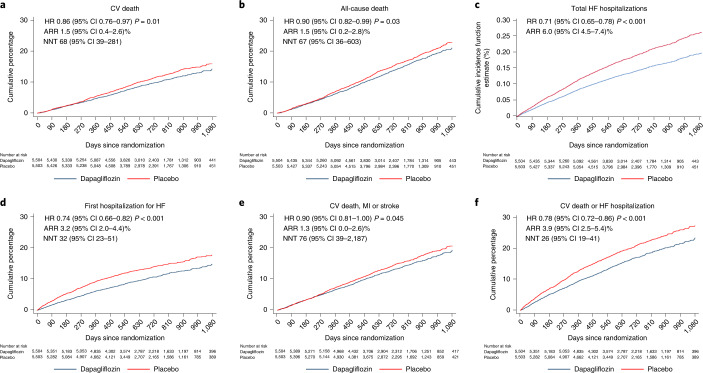
Effect of dapagliflozin on key clinical outcomes in pooled DAPA-HF and DELIVER dataset. **a**–**f**, Incidence of: death from CV causes (**a**); death from all causes (**b**); the total number of hospital admissions for HF (**c**); time to first hospital admission for HF (**d**); death from CV causes, MI or stroke (**e**); and death from CV causes or hospital admission for HF (**f**), according to randomized therapy. Participants randomized to dapagliflozin are shown in blue and those randomized to placebo in red. All figures are Kaplan–Meier curves with an HR and 95% CI estimated from Cox’s model with two-sided *P* values except for the total number of hospital admissions for HF, which was plotted using the Gosh and Lin method accounting for death from CV causes (the RR is estimated from the joint frailty model with a two-sided *P* value). No adjustment for multiple comparisons was made. NNT indicates the number of patients who need to be treated over the median duration of follow-up to prevent one event (of the type in each panel). An NNT could not be calculated for the total number of hospital admissions for HF because this was an episode-based rather than a patient-based analysis (that is, patients may have had more than one hospital admission). ARRs and NNTs are shown with a 95% CI.

**Fig. 2:**
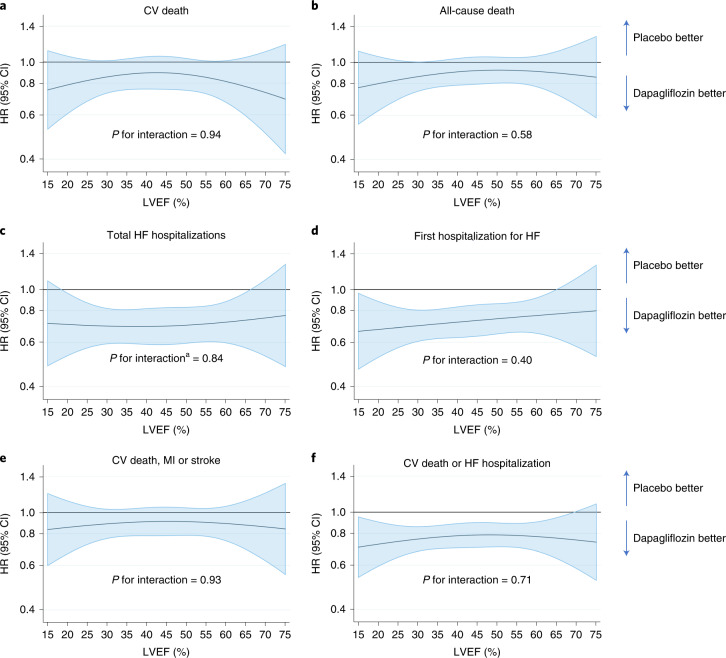
Effect of dapagliflozin on clinical outcomes across the range of ejection fraction. **a**–**f**, Effect of dapagliflozin on: death from CV causes (**a**); death from all causes (**b**); the total number of hospital admissions for HF (**c**); time to first hospital admission for HF (**d**); death from CV causes, MI or stroke (**e**); and death from CV causes or hospital admission for HF (**f**), according to baseline LVEF. The horizontal blue line shows the continuous HR across the range of LVEF and the shaded area around this line represents the 95% CI from Cox’s model. The overall effect of treatment in the pooled population is shown in each panel as an HR (95% CI) with the two-sided *P* value from Cox’s model for Wald’s test of interaction between treatment and LVEF. No adjustment for multiple comparisons was made. ^a^Restricted cubic spline and interaction *P* value derived from LWYY model for total HF hospitalization.

The rate of each pre-specified outcome was lower in the dapagliflozin group (Fig. 1). In the overall population, dapagliflozin reduced the risk of death from CV causes with an HR of 0.86 (95% CI 0.76–0.97), *P* = 0.01. There was no evidence of effect modification by ejection fraction examined as either a categorical (Table 2 and Fig. 2) or a continuous variable (*P* for interaction = 0.63 and 0.94, respectively).

**Table 2:** Clinical outcomes according to ejection fraction category and randomized therapy

	≤30%		>30 and ≤37%		>37 and ≤44%		>44 and ≤51%		>51 and ≤60%		>60%		Pooled cohort	
	Placebo	Dapa	Placebo	Dapa	Placebo	Dapa	Placebo	Dapa	Placebo	Dapa	Placebo	Dapa	Placebo	Dapa
**CV death**														
*n*/*N*	154/1,099	126/1,062	74/785	65/799	106/900	93/963	109/947	96/915	96/1,054	102/1,088	68/718	43/677	607/5,503	525/5,504
Rate per 100 patient years (95% CI)	9.9 (8.5–11.6)	8.4 (7.0–10.0)	6.4 (5.1–8.1)	5.6 (4.4–7.1)	6.8 (5.6–8.2)	5.4 (4.4–6.6)	5.2 (4.3–6.3)	4.7 (3.9–5.8)	4.1 (3.3–4.9)	4.2 (3.4–5.1)	4.3 (3.4–5.4)	2.8 (2.1–3.8)	5.9 (5.4–6.4)	5.1 (4.6–5.5)
HR (95% CI)	0.85 (0.67–1.07)		0.86 (0.61–1.20)		0.81 (0.61–1.07)		0.91 (0.69–1.20)		1.02 (0.77–1.34)		0.68 (0.47–1.00)		0.86 (0.76–0.97)	
**All-cause death**														
*n*/*N*	172/1,099	145/1,062	94/785	78/799	147/900	137/963	169/947	153/915	159/1,054	169/1,088	114/718	91/677	855/5,503	773/5,504
Rate per 100 patient years (95% CI)	11.1 (9.5–12.9)	9.6 (8.2–11.3)	8.1 (6.6–10.0)	6.7 (5.3–8.3)	9.4 (8.0–11.0)	7.9 (6.7–9.4)	8.0 (6.9–9.3)	7.5 (6.4–8.8)	6.7 (5.7–7.8)	6.9 (5.9–8.0)	7.2 (6.0–8.6)	6.0 (4.9–7.4)	8.3 (7.7–8.8)	7.4 (6.9–8.0)
HR (95% CI)	0.87 (0.70–1.09)		0.81 (0.60–1.09)		0.86 (0.68–1.08)		0.94 (0.75–1.17)		1.02 (0.82–1.27)		0.86 (0.65–1.13)		0.90 (0.82–0.99)	
**Total HF hospitalizations**														
*n*/*N*	274/1,099	179/1,062	128/785	111/799	171/900	119/963	219/947	179/915	250/1,054	165/1,088	134/718	95/677	1176/5,503	848/5,504
Rate per 100 patient years (95% CI)	17.8 (15.8–20.0)	11.9 (10.3–13.8)	11.2 (9.4–13.3)	9.5 (7.9–11.5)	10.9 (9.4–12.7)	6.9 (5.8–8.38)	10.5 (9.2–12.0)	8.8 (7.6–10.2)	10.6 (9.4–12.0)	6.8 (5.8–7.9)	8.5 (7.1–10.0)	6.3 (5.2–7.7)	11.4 (10.8–12.1)	8.2 (7.7–8.8)
RR (95% CI)	0.66 (0.54–0.80)		0.84 (0.64–1.09)		0.62 (0.48–0.79)		0.84 (0.68–1.03)		0.63 (0.51–0.77)		0.77 (0.59–1.02)		0.71 (0.65–0.78)	
**HF hospitalization**														
*n*/*N*	179/1,099	118/1,062	93/785	74/799	102/900	88/963	131/947	107/915	150/1,054	107/1,088	81/718	66/677	736/5,503	560/5,504
Rate per 100 patient years (95% CI)	12.4 (10.7–14.4)	8.2 (6.9–9.8)	8.6 (7.0–10.5)	6.6 (5.3–8.3)	6.9 (5.7–8.4)	5.4 (4.4–6.6)	6.7 (5.7–8.0)	5.6 (4.6–6.7)	6.9 (5.8–8.1)	4.6 (3.8–5.6)	5.4 (4.4–6.8)	4.6 (3.6–5.9)	7.7 (7.1–8.2)	5.7 (5.2–6.2)
HR (95% CI)	0.66 (0.52–0.83)		0.76 (0.56–1.03)		0.78 (0.59–1.04)		0.83 (0.64–1.07)		0.66 (0.51–0.84)		0.88 (0.64–1.22)		0.74 (0.66–0.82)	
**CV death, MI or stroke**														
*n*/*N*	176/1,099	153/1,062	99/785	88/799	133/900	125/963	150/947	130/915	139/1,054	153/1,088	91/718	66/677	788/5,503	715/5,504
Rate per 100 patient years (95% CI)	11.6 (10.0–13.4)	10.3 (8.8–12.1)	8.8 (7.2–10.7)	7.7 (6.2–9.5)	8.7 (7.3–10.3)	7.4 (6.2–8.9)	7.3 (6.3–8.6)	6.6 (5.5–7.8)	6.0 (5.1–7.1)	6.4 (5.5–7.5)	5.8 (4.7–7.2)	4.5 (3.5–5.7)	7.8 (7.3–8.4)	7.1 (6.6–7.6)
HR (95% CI)	0.90 (0.72–1.11)		0.86 (0.65–1.15)		0.87 (0.68–1.11)		0.89 (0.70–1.13)		1.07 (0.85–1.34)		0.77 (0.56–1.06)		0.90 (0.81–1.00)	
**CV death or HF hospitalization**														
*n*/*N*	271/1,099	203/1,062	141/785	117/799	183/900	153/963	195/947	173/915	209/1,054	175/1,088	129/718	92/677	1128/5,503	913/5,504
Rate per 100 patient years (95% CI)	18.8 (16.7–21.2)	14.1 (12.3–16.2)	13.0 (11.0–15.3)	10.4 (8.7–12.5)	12.5 (10.8–14.4)	9.3 (8.0–10.9)	10.0 (8.7–11.5)	9.0 (7.8–10.4)	9.6 (8.4–11.0)	7.5 (6.5–8.7)	8.7 (7.3–10.3)	6.4 (5.2–7.9)	11.7 (11.1–12.4)	9.2 (8.7–9.9)
HR (95% CI)	0.75 (0.63–0.90)		0.79 (0.62–1.01)		0.75 (0.61–0.93)		0.90 (0.73–1.10)		0.77 (0.63–0.95)		0.77 (0.59–1.00)		0.78 (0.72–0.86)	

HRs and the 95% CI are estimated from Cox’s model and the rate ratio (RR) and 95% CI are estimated from a joint frailty model with death from CV causes as a competing event.

Dapa, dapagliflozin.

**Extended Data Fig. 2:**
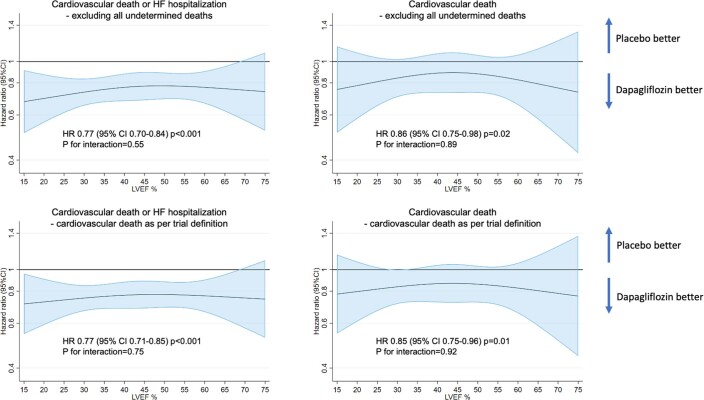
Effect of dapagliflozin on clinical outcomes across the range of NT-proBNP. Effect of dapagliflozin on death from cardiovascular causes (CV death) and CV death or hospitalisation for heart failure (HF hospitalisation) where the definition of CV death used excluded undetermined deaths from the definition of CV death (top two panels) and according to the original trial definitions (that is, including undetermined deaths in DAPA-HF and excluding undetermined deaths in DELIVER) (bottom two panels). The horizontal blue line shows the continuous hazard ratio (HR) across the range of left ventricular ejection fraction (LVEF) and the shaded area around this line represents the 95% confidence interval (95%CI) estimated from a Cox model. The overall effect of treatment in the pooled population is shown in each panel as a HR (95%CI) with the two-sided p-value estimated from a Cox model for the Wald test of interaction between treatment and LVEF. No adjustment for multiple comparisons was made.

In sensitivity analyses, the results were unchanged when undetermined deaths were excluded from the definition of death from CV causes or if the definition of death from CV causes used in each trial was examined (Extended Data Fig. 2). The absolute risk reduction (ARR) was 1.5% (95% CI 0.4–2.6%) and the number needed to treat (NNT) over the median follow-up was 68 (95% CI 39–281).

The risk of death from any cause was also reduced (HR 0.90 (95% CI 0.82–0.99); *P* = 0.03) with no evidence of an interaction between ejection fraction and treatment, whether ejection fraction was analyzed by category (*P* for interaction = 0.79) or as a continuous variable (*P* for interaction = 0.58). The ARR was 1.5% (95% CI 0.2–2.8%) and the NNT over the median follow-up was 67 (95% CI 36–603).

Dapagliflozin reduced the risk of total (that is, first and subsequent) hospital admissions for HF (RR 0.71 (95% CI 0.65–0.78), *P* < 0.001) and there was no evidence of a treatment interaction with ejection fraction, whether analyzed by category (*P* for interaction = 0.62) or as a continuous variable (*P* for interaction = 0.84). The pre-specified supportive analysis of time to first hospital admission showed a consistent benefit of dapagliflozin (HR 0.74 (95% CI 0.66–0.82); *P* < 0.001). The ARR was 3.2% (95% CI 2.0–4.4%) and the NNT over the median follow-up was 32 (95% CI 23–51).

Applying the overall relative risk reduction to the placebo group event rate gave an NNT (95% CI) to prevent a death from CV causes in patients with reduced, mildly reduced and preserved ejection fractions of 61 (37–246), 59 (35–237) and 76 (46–309), respectively. The corresponding NNTs for a first hospitalization for HF were 28 (21–41), 30 (24–45) and 29 (23–43) and, for death from any cause, 72 (39–764), 56 (31–593) and 64 (35–684), respectively.

Compared with placebo, dapagliflozin also reduced the incidence of the MACE composite of death from CV causes, MI or stroke, although this effect was of borderline statistical significance (HR 0.90 (5% CI 0.81–1.00); *P* = 0.045). Again, there was no interaction between ejection fraction and the effect of treatment whether analyzed categorically (*P* for interaction = 0.72) or as a continuous measure (*P* for interaction = 0.93). The ARR was 1.3% (95% CI 0.0–2.6%) and the NNT over the median follow-up was 76 (95% CI 39–2187).

To address the possible attenuation of treatment benefit at higher ejection fractions reported in the EMPEROR trials^12^, we examined the effect of dapagliflozin on the primary composite endpoint used in those trials, that is, time to the first occurrence of hospital admission for worsening HF or death from CV causes. Dapagliflozin reduced the risk of this outcome by 22% (HR 0.78 (95% CI 0.72–0.86); *P* < 0.001) (Table 2 and Fig. 2). The benefit appeared consistent across ejection fraction categories, with the test for interaction between ejection fraction and the effect of dapagliflozin giving a *P* value of 0.82 (Table 2). Inspection of the restricted cubic spline showed that the HR was below unity across the full range of ejection fraction, with the upper 95% CI around the HR crossing unity only at the extreme ends of the range (at around 9% and 70%, respectively), probably due to the small number of patients with either a very high or a very low ejection fraction. The *P* value for the test of interaction was 0.71. In sensitivity analyses, the results were unchanged if undetermined deaths were excluded from the definition of death from CV causes or if the definition from the individual trials was used (Extended Data Fig. 2).

### Effect of dapagliflozin in the pre-specified subgroups

**Fig. 3:**
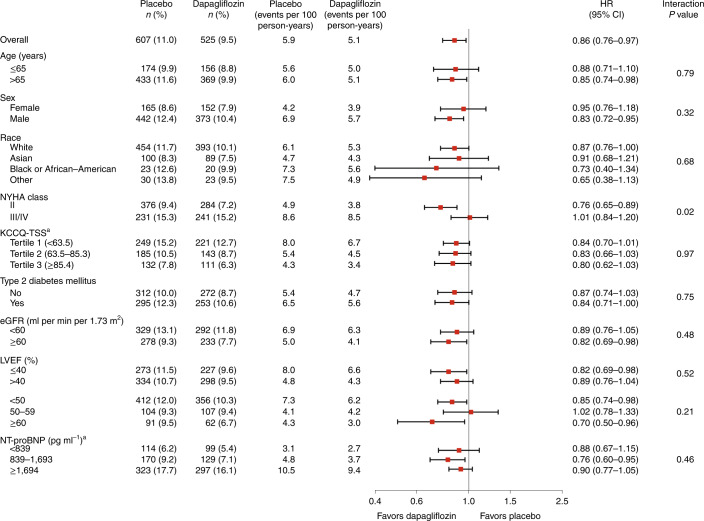
Effect of randomized treatment on CV death according to the pre-specified subgroups. Estimates are HRs with error bars representing 95% CIs from Cox’s model and a two-sided *P* value for interaction from Wald’s test of Cox’s model. No adjustment for multiple comparisons was made. ^a^Not a pre-specified subgroup.

**Fig. 4:**
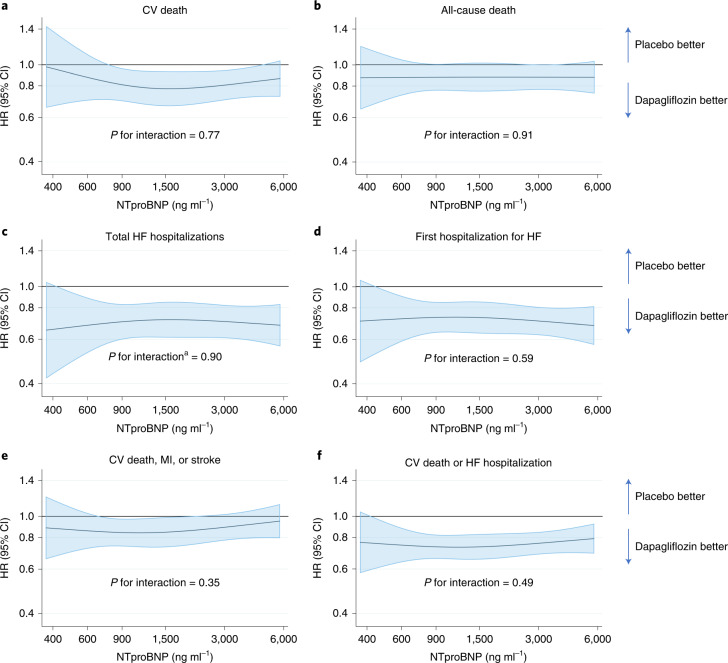
Effect of dapagliflozin on clinical outcomes across the range of NT-proBNP. **a**–**f**, Effect of dapagliflozin on: death from CV causes (**a**); death from all causes (**b**); the total number of hospital admissions for HF (**c**); time to first hospital admission for HF (**d**); death from CV causes, MI or stroke (**e**); and death from CV causes or hospital admission for HF (**f**), according to baseline NT-proBNP level. The horizontal blue line shows the continuous HR across the range of NT-proBNP levels at baseline and the shaded area around this line represents the 95% CI from Cox’s model. The overall effect of treatment in the pooled population is shown in each panel as an HR (95% CI) with the two-sided *P* value for Wald’s test of interaction between treatment and NT-proBNP level from Cox’s model. No adjustment for multiple comparisons was made. ^a^Restricted cubic spline and interaction *P* value derived from LWYY model for total HF hospitalization.

The effect of dapagliflozin on CV death was consistent across the pre-specified subgroups except for NYHA class, where the benefit seemed to be less in patients who were in a worse functional class (Fig. 3). To determine whether this interaction was likely to be true or to reflect the play of chance, we also examined the interaction between the KCCQ-TSS score and the effect of dapagliflozin on death from CV causes in a post-hoc subgroup analysis and found that the interaction was not significant (Fig. 3). We also conducted a post-hoc subgroup analysis using NT-proBNP as a continuous measure modeled as a restricted cubic spline and found no evidence of a difference in the effect of dapagliflozin by baseline NT-proBNP levels for any of the outcomes (Fig. 4).

## Discussion

In a patient-level pooled meta-analysis of 11,007 participants in DAPA-HF and DELIVER^1,2^, compared with placebo, dapagliflozin 10 mg once daily reduced the risk of each of the pre-specified endpoints, that is, death from CV causes (by 14%), death from any cause (by 10%), total (first and repeat) hospital admissions for HF (by 29%) and the composite of death from CV causes, MI or stroke (by 10%), in patients with HF, with no evidence of heterogeneity of the benefit across the range of ejection fractions.

The original reason for planning a pooled analysis of DAPA-HF and DELIVER was to provide a more statistically robust estimate of the effect of dapagliflozin on outcomes that the individual trials had limited power to examine. Of particular interest was death from CV causes, and death from any cause, as neither trial was powered to show a modest benefit of dapagliflozin on these endpoints, which could still be clinically important. There was a significant benefit of dapagliflozin on death from CV causes in DAPA-HF (HR 0.82 (95% CI 0.69–0.98)) but the present analysis provides a more reliable and precise estimate of the effect of treatment (HR 0.86 (95% CI 0.76–0.97)). Using the pooled analysis of DAPA-HF and DELIVER, the number of patients with HF who needed to be treated (NNT) for a median of 22 months to prevent one death from CV causes was 68 (95% CI 39–281). The conclusion for death from any cause was similar, with a modest-sized benefit that was statistically significant. The reduction in MACE was of borderline statistical significance. However, the beneficial effect on hospital admissions for HF was substantial, as was observed in the individual trials with SGLT2 inhibitors in HF. As a result, our pooled analysis demonstrates the large and generally consistent effect of dapagliflozin on this key outcome in patients with HF, irrespective of ejection fraction phenotype. Although there was a nominally significant interaction between NYHA class and the effect of dapagliflozin, NYHA class and KCCQ-TSS score were dissociated across the spectrum of LVEF at baseline and the effect of dapagliflozin was consistent across the range of KCCQ-TSS scores included.

The second and potentially more important reason to conduct the pooled analysis of DAPA-HF and DELIVER was to address the surprising findings of a pooled analysis of the EMPEROR trials, which appeared to show that the size of the reduction in risk of hospital admission for worsening HF with empagliflozin declined as LVEF increased, with an apparent loss of effect in patients with an ejection fraction in the region of 60–65%^12^. Although this attenuation of benefit with increasing ejection fraction has been shown repeatedly with treatments acting on neurohumoral pathways^9–11^, it was not expected with SGLT2 inhibitors. We did not find any attenuation of the effect of dapagliflozin with increasing ejection fraction for any of the outcomes of interest, including the EMPEROR primary endpoint of first hospitalization for HF or death from CV causes, with consistently nonsignificant tests of interaction between ejection fraction and the effect of treatment. We also found no interaction according to baseline NT-proBNP level as a measure of neurohumoral activation, although the minimum NT-proBNP inclusion threshold was 300 pg ml^−1^ and some patients with HF with preserved ejection fraction (HFpEF) have levels below this^13^.

The seemingly contrary findings of the pooled EMPEROR trials^11^ and the present analysis are not explained by the distribution of ejection fraction, which was similar in each. The pooled analysis of the dapagliflozin trials included 1,289 more patients than the equivalent analysis of the empagliflozin trials. Therefore, we think that the findings of the present analysis are probably more reliable and those of the EMPEROR analysis may have been spurious, given that they were unexpected and observed in a post-hoc analysis, and whether there was a significant ejection fraction-by-treatment interaction was uncertain. However, we cannot conclude that this is definitely the case and our findings cannot necessarily be generalized to other SGLT2 inhibitors. In addition, in a randomized trial testing the effect of dapagliflozin on symptoms and functional capacity in patients with HFpEF, there was no heterogeneity of treatment effect according to ejection fraction^14^.

Our findings have clinical implications. Currently, except for diuretics, treatment for HF depends on knowledge of ejection fraction, the measurement of which may not be immediately available, especially where there are limited healthcare resources or geographical or other barriers to obtaining specialist care. The consistency of benefit of SGLT2 inhibitors across the range of ejection fraction, the rapidity with which benefit is obtained^15,16^, the lack of requirement for titration of dose and the excellent safety profile suggest that this treatment could be initiated while waiting for ejection fraction to be measured. A modeling exercise suggested that first-line treatment with an SGLT2 inhibitor maximizes the benefit of evidence-based treatments in patients with reduced ejection fraction^17^. Moreover, no other treatment for patients with mildly reduced or preserved ejection fraction has the same strength of evidence as SGLT2 inhibitors^18^.

Our study has several limitations. LVEF was reported by investigators and was not measured in a core laboratory. As commonly found, there was digit preference in the ejection fraction measurements reported. However, we minimized this effect by examining all outcomes with ejection fraction modeled as a continuous variable and using categories that utilized mid-point ranges rather than whole numbers. We also had a minimum NT-proBNP inclusion threshold of 300 pg ml^−1^ in DELIVER and it is known that some patients with HFpEF have an NT-proBNP level below this value. Consequently, we cannot be sure about the generalizability of our findings to these patients.

Our analysis demonstrates that, in patients with HF, dapagliflozin led to significant reductions in the risk of death from CV causes and any cause, as well as MACE, irrespective of LVEF. There was a larger reduction in total hospital admissions for HF than in death, which was also consistent across the range of ejection fractions. Most patients with HF, regardless of ejection fraction, are likely to benefit from treatment with an SGLT2 inhibitor, although the ARR is somewhat smaller in patients with higher compared with lower ejection fractions. This analysis supports a recommendation that treatment with dapagliflozin can be initiated in patients with a clinical diagnosis of HF and no contraindications, even if a measurement of ejection fraction is awaited.

## Methods

### Patient-level pooled meta-analysis of DAPA-HF and DELIVER

The design and results of the DAPA-HF (clinicaltrials.gov identifier NCT03036124) and DELIVER (clinicaltrials.gov identifier NCT03619213) trials have been published^1,2,6,7^. Each enrolled patient had a diagnosis of HF, functional limitation and elevated natriuretic peptides. The principal difference between the two trials was that patients with an ejection fraction ≤40% were randomized in DAPA-HF and those with an ejection fraction >40% in DELIVER. In both trials, patients were randomized to dapagliflozin at a dose of 10 mg once daily, or a matching placebo, in addition to standard care. The ethics committees of the participating institutions approved the protocols and all patients gave written informed consent.

### Trial patients

Patients in NYHA functional classes II–IV, with an LVEF ≤ 40% and an elevated NT-proBNP level, were eligible for DAPA-HF. Participants were also required to receive guideline-recommended treatments for HF with reduced ejection fraction. The main exclusions to enrollment were a history of type 1 diabetes mellitus, hypotension causing symptoms or a systolic blood pressure <95 mmHg and an eGFR <30 ml per min per 1.73 m^2^.

Patients in NYHA functional classes II–IV, with an LVEF > 40% and an elevated NT-proBNP level were eligible for DELIVER. Participants were also required to have evidence of structural heart disease (defined as either left atrial enlargement or left ventricular hypertrophy). All patients in DELIVER had to be receiving at least intermittent diuretic therapy, but no specific background therapy was mandated during the trial. The key exclusion criteria were similar to those in DAPA-HF, although the eGFR threshold was lower in DELIVER (25 ml per min per 1.73 m^2^).

In both trials, patients with and without type 2 diabetes were randomized and randomization in both trials was stratified by type 2 diabetes status.

### Outcomes

Both trials were event driven and had the same primary endpoint, which was a composite of the time to the first occurrence of worsening HF or death from a CV cause. Worsening HF was defined as unplanned hospital admission for HF or an urgent visit for worsening HF resulting in the administration of an intravenous diuretic.

In the original ‘regulatory’ statistical analysis plan for the meta-analysis (dated 2 August 2019), a pre-specified hierarchy of endpoints was provided with control of alpha (see Statistical analysis below). The endpoints were: death from CV causes; death from any cause; total (that is, first and repeat) hospital admissions for HF (with an additional supportive analysis of time to the first occurrence of hospital admissions for HF, outside alpha control); and the composite of death from CV causes, MI or stroke (MACEs). As a result of the possible attenuation of the benefit of SGLT2 inhibition at higher ejection fractions reported in the EMPEROR trials^12^ (as described in the introduction), we also examined the composite outcome used in the EMPEROR trials, that is, time to the first occurrence of hospital admission for worsening HF or death from CV causes in our analyses.

The original statistical analysis plan stated that the consistency of the effect of dapagliflozin on CV death would be examined in a limited number of subgroups defined by age (≤65, >65 years), sex (male, female), race (white, black or African, Asian, other), NYHA class at enrollment (II, III/IV), LVEF at enrollment (≤40, >40%), diagnosis of type 2 diabetes mellitus at baseline (yes, no) and eGFR at baseline (<60 or ≥60 ml per min per 1.73 m^2^). As described below, additional ejection fraction subgroups were included in an updated statistical analysis plan.

In DAPA-HF, the definition of a CV death included any death not judged to have a non-CV cause, that is, deaths where the cause could not be determined. By contrast, in DELIVER, deaths in which the cause could not be determined were excluded from the definition of death from CV causes. In the pre-specified statistical analysis plan, the definition of death from CV causes included deaths of undetermined causes. However, we also conducted a sensitivity analysis using the definitions originally employed in the individual trials.

MI and stroke were adjudicated in DAPA-HF but not in DELIVER, where serious adverse event reports were used to ascertain these outcomes.

The ‘academic’ statistical analysis plan, dated 30 March 2022, stated that additional LVEF subgroups in addition to those described in the DELIVER statistical analysis plan (that is, ≤ 49%, 50–59%, ≥60%) would be considered to limit digit preference and the effects of treatment would be examined using LVEF as a continuous measure.

### Statistical analysis

Before pooling DELIVER and DAPA-HF, between-trial heterogeneity was tested as pre-specified using *Q* and *I*^2^ statistics. There was little evidence of heterogeneity for the effect of treatment on the primary outcome, that is, death from CV causes (*Q* = 0.47, *P* = 0.50 and *I*^2^ = 0%).

The estimand was formulated as treatment with dapagliflozin would reduce the risk of: death from CV causes; death from any cause; total (that is, first and repeat) hospital admissions for HF; and the composite of death from CV causes, MI or stroke (MACEs) in adults with HF, irrespective of exposure, treatment discontinuation or concomitant treatment. To control the family-wise error rate at the 5% alpha level, a fixed sequence procedure was used with the testing procedure continued down the hierarchy, if the preceding endpoint was rejected at the 5% alpha level.

Baseline characteristics were summarized as means (s.d.), median (IQRs) or percentages and described across groups according to ejection fraction. Ejection fraction was normally distributed but demonstrated digit preference and, to account for this, sextiles were used to describe the distribution of baseline characteristics. Cochrane, Armitage and Cuzick’s tests were used to examine trends across ejection fraction quantiles. Rates were calculated using the total number of events divided by the person-years of follow-up and expressed as a rate per 100 person-years. Cox’s models included randomized therapy and were stratified by diabetes status at enrollment and trial (DAPA-HF or DELIVER). To account for the clustering within trials, a variable denoting the trial was used as a stratification variable in the model, to indicate that different trial populations are exposed to different baseline risks^19^. The effect of therapy according to ejection fraction was tested in Cox’s models by entering an interaction term between randomized therapy and ejection fraction as a continuous variable modeled as a restricted cubic spline. Three knots were chosen (ejection fraction of 6%, 45% and 84%) after examining the Akaike information criterion (AIC) for different numbers of knots, and the spline with the lowest AIC was chosen. All models used the full range of ejection fraction values. The interaction was represented graphically showing the HR for the effect of dapagliflozin against placebo across the range of ejection fraction. Total HF hospitalizations were analyzed by a joint frailty model with CV death treated as a competing risk^20^. The model included the treatment term and adjustment for previous hospital admission for HF, diabetes status at enrollment and trial (DAPA-HF or DELIVER). The nonparametric estimates of the marginal mean of the cumulative number of total hospital admissions for HF over time were calculated allowing for death as a terminal event, and the estimates were plotted according to the approach of Ghosh and Lin^21^. To examine the interaction between the effect of dapagliflozin on each CV death and total hospital admissions for HF, a spline term for ejection fraction, as outlined above, was entered into an extension of the proportional hazards model for recurrent events as described by the Lin–Wei–Yang–Ying (LWYY) model, which is a semiparametric proportional rates model^22^. The continuous RR interaction term was then plotted.

All analyses were conducted using Stata v.17.0 and SAS v.9.4. There were no missing data for the variables used in the models and missing follow-up data were handled by censoring at the time of the assessment for potential endpoints. Few patients in either trial had an incomplete follow-up. A *P* < 0.05 was considered statistically significant.

### Reporting summary

Further information on research design is available in the Nature Research Reporting Summary linked to this article.

## Online content

Any methods, additional references, Nature Research reporting summaries, extended data, supplementary information, acknowledgements, peer review information; details of author contributions and competing interests; and statements of data and code availability are available at 10.1038/s41591-022-01971-4.

## Supplementary information

Supplementary Information Statistical analysis plans combined as one PDF.

Reporting Summary

## Data Availability

Data underlying the findings described in this manuscript may be obtained following AstraZeneca’s data-sharing policy described at https://astrazenecagrouptrials.pharmacm.com/ST/Submission/Disclosure.

## Extended data

Extended data are available for this paper at 10.1038/s41591-022-01971-4.

## Code availability

Code for the analyses in this manuscript can be shared on request to the corresponding author if data have been released following the data-sharing policy described at https://astrazenecagrouptrials.pharmacm.com/ST/Submission/Disclosure.
