# Supplementary material for: Dapagliflozin across the range of ejection fraction in patients with heart failure: a patient-level, pooled meta-analysis of DAPA-HF and DELIVER
Source: Nat Med. 2022 Aug 27;28(9):1956–64. doi: 10.1038/s41591-022-01971-4 (PMC9499855; doi:10.1038/s41591-022-01971-4)
Supplement: Supplementary file 1 — Statistical analysis plans combined as one PDF. [file 41591_2022_1971_MOESM1_ESM.pdf]

nature portfolio

https://doi.org/10.1038/s41591-022-01971-4

Supplementary information

Dapagliflozin across the range of ejection fraction in patients with heart failure: a patient-level, pooled meta-analysis of DAPA-HF and DELIVER

In the format provided by the authors and unedited

Addendum Statistical Analysis Plan	
Drug substance	Dapagliflozin
Date	02 August 2019

Meta-Analysis of Dapagliflozin data in Heart Failure

Addendum to Statistical Analysis Plan of DELIVER study

TABLE OF CONTENTS

TITLE PAGE....1
TABLE OF CONTENTS....2
1 DAPAGLIFLOZIN CLINICAL DEVELOPMENT PROGRAM....4
2 OBJECTIVES....8
3 INTEGRATION OF EFFICACY AND SAFETY DATA....9
4 EFFICACY AND SAFETY VARIABLES....11
4.1 Endpoints to be included in the integrated analyses....11
4.2 Definition of Subgroups....12
4.3 Safety Evaluations....12
5 ANALYSIS STRATEGY....12
5.1 General Principles....12
5.2 Analysis Population....13
5.3 Subject Characterization....13
5.3.1 Subject Identification....13
5.3.2 Subject Disposition....13
5.3.3 Demographic and Baseline Characteristics....13
5.4 Efficacy Analyses....14
5.4.1 Hypotheses and Confirmatory Testing Procedure....14
5.4.2 Time-to-Event Endpoints....14
5.4.3 Recurrent HF hospitalizations....15
5.4.4 Proportion of patients with a new diagnosis of T2DM in non-diabetic subpopulation at baseline....16
6 POWER CALCULATION....17
7 REFERENCES....18

LIST OF ABBREVIATIONS AND DEFINITION OF TERMS

Abbreviation or special term	Explanation
CEA	Clinical Event Adjudication
CKD	Chronic kidney disease
CV	Cardiovascular
CVD	Cardiovascular disease
eGFR	Estimated glomerular filtration rate
ESRD	End stage renal disease
EU	European Union
FAS	Full analysis set
HbA1c	Glycated hemoglobin
HF	Heart failure
HFpEF	Heart failure with preserved ejection fraction
HFrEF	Heart failure with reduced ejection fraction
HR	Hazard ratio
IND	Investigational new drug
KM	Kaplan-Meier (estimates)
LVEF	Left ventricular ejection fraction
MACE	Major adverse cardiovascular events (in this study: cardiovascular death, ischemic stroke and myocardial infarction)
MI	Myocardial infarction
NCT	National clinical trial
NDA	New drug application
NYHA	New York Heart Association
PACD	Primary analysis censoring date
SAP	Statistical analysis plan
SD	Standard deviation
T1DM	Type 1 diabetes mellitus
T2DM	Type 2 diabetes mellitus
UACR	Urine albumin creatinine ratio
UK	United Kingdom
US	United States
WoC	Withdrawal of consent

1 DAPAGLIFLOZIN CLINICAL DEVELOPMENT PROGRAM

FARXIGA® (dapagliflozin) is a sodium glucose co-transporter 2- (SGLT2)-inhibitor that is currently indicated as an adjunct to diet and exercise to improve glycemic control in adults with type 2 diabetes mellitus (T2DM) for which it is approved in more than 90 countries, including the European Union (EU) since November 2012, and in the United States (US) since January 2014 (FARXIGA NDA 202293). Dapagliflozin can be given as monotherapy or in combination with other medical products indicated for the treatment of T2DM. In addition to patients with T2DM, dapagliflozin has also been studied in patients with T2DM and established cardiovascular disease (CVD), in patients with T2DM and cardiovascular (CV) risk factors, and in patients with type 1 diabetes mellitus (T1DM).

The ongoing clinical development program evaluates the effect of dapagliflozin on mortality, morbidity (hospitalization), and physical function endpoints in patients with an established diagnosis of chronic heart failure (HF) with reduced ejection fraction (HFrEF), and in patients with an established diagnosis of HF with preserved ejection fraction (HFpEF). The heterogeneity of the HFpEF population (Lam et al 2018), compared with the HFrEF population, is thought to be a reason why there are currently no effective treatment options for HFpEF, despite recent advances in the management and treatment of chronic HFrEF. In addition, dapagliflozin is also being studied patients with chronic kidney disease (CKD) with or without history of HF. The intention of AstraZeneca is to seek marketing approval for the use of dapagliflozin in patients with chronic HF and also patients with CKD.

Table 1 presents a summary of more recent clinical studies that include patients with chronic heart failure, or with either established CVD, or CV risk factors.

Table 1 Clinical trials of the AstraZeneca dapagliflozin clinical development program

Trial ID<br>NCT number<br>(Study acronym)	IND/NDA	Trial title	Dosing regimen	Trial population	Planned enrollment/ trial start
D1693C00001<br>NCT 01730534<br>(DECLARE-TIMI 58)[a]	FARXIGA<br>NDA 202293 (s-018)<br>XIGDUO XR<br>NDA 205649 (s-011)	Dapagliflozin Effect on CardiovascuLAR Events: A Multicenter, Randomized, Double-Blind, Placebo-Controlled Trial to Evaluate the Effect of Dapagliflozin 10 mg Once Daily on the Incidence of Cardiovascular Death, Myocardial Infarction or Ischemic Stroke in Patients with Type 2 Diabetes	Dapagliflozin 10 mg versus<br>Placebo<br>Randomization 1:1	• Males and females<br>• age ≥40 years<br>• Diagnosed with T2DM<br>• High Risk for Cardiovascular events	**Study completed**.<br>N=17,160<br>sNDAs (s-018 and s-011) under review with PDUFA date of 18 October 2019[a]
D1699C00001<br>NCT 03036124<br>(DAPA-HF)	IND 130631	Study to Evaluate the Effect of Dapagliflozin on the Incidence of Worsening Heart Failure or Cardiovascular Death in Patients with Chronic Heart Failure with Reduced Ejection Fraction.<br>Short title: Dapagliflozin And Prevention of Adverse outcomes in Heart Failure	Dapagliflozin 10 mg versus<br>Placebo<br>Randomization 1:1	• Males and females<br>• age ≥18 years,<br>• with heart failure (NYHA II-IV)<br>• with T2DM or without diabetes	**Study clinically completed.**<br>N=4744<br>08 February 2017

Trial ID<br>NCT number<br>(Study acronym)	IND/NDA	Trial title	Dosing regimen	Trial population	Planned enrollment/ trial start
D169EC00001<br>NCT 03877224<br>(DETERMINE-preserved)<br>D169EC00002<br>NCT 03877237<br>(DETERMINE-reduced)	IND 136809<br><br><br>IND 130631	An International, Multicentre, Parallel-group, Randomised, Double-blind, Placebo-controlled, Phase III Study Evaluating the effect of Dapagliflozin on Exercise Capacity in Heart Failure Patients with preserved Ejection Fraction (HFpEF[b]) or reduced Ejection Fraction (HFrEF[c])<br>Carried out as 2 independent trials:<br>**DETERMINE-preserved**: Dapagliflozin EffecT on ExeRcise capacity using a 6-MINutE walk test in patients with heart failure with preserved ejection fraction (HFpEF)<br>AND<br>**DETERMINE-reduced**: Dapagliflozin EffecT on ExeRcise capacity using a 6-MINutE walk test in patients with heart failure with reduced ejection fraction (HFrEF)	Dapagliflozin 10 mg versus<br>Placebo<br>Randomization 1:1	• Males and females<br>• age ≥40 years<br>• with heart failure (NYHA II-IV)<br>• with T2DM or without diabetes	N=400 (HFpEF)<br>04 April 2019<br>N=300 (HFrEF)<br>09 April 2019

Trial ID<br>NCT number<br>(Study acronym)	IND/NDA	Trial title	Dosing regimen	Trial population	Planned enrollment/ trial start
D169CC00001<br>NCT 03619213<br>(DELIVER)	IND 136809	An International, Double-blind, Randomised, Placebo-Controlled Phase IIIb Study to Evaluate the Effect of Dapagliflozin on Reducing CV Death or Worsening Heart Failure in Patients with Heart Failure with Preserved Ejection Fraction (HFpEF)	Dapagliflozin10 mg versus<br>Placebo<br>Randomization 1:1	• Males and females<br>• age ≥40 years<br>• with HFpEF<br>• with T2DM or without diabetes	N=4700<br>27 August 2018
D169AC0001<br>NCT 03036150<br>(DAPA-CKD)	IND 130647	A Study to Evaluate the Effect of Dapagliflozin on Renal Outcomes and Cardiovascular Mortality in Patients with Chronic Kidney Disease	Dapagliflozin10 mg versus<br>Placebo<br>Randomization 1:1	• Males and females<br>• age ≥18 years<br>• with CKD[d]<br>• with albuminuria[e]<br>• with T2DM or without diabetes	N=4000<br>02 February 2017

[a] Study D1693C00001 (DECLARE-TIMI 58) was a post marketing requirement study requested by the FDA: PMR 2121-5; DECLARE-TIMI 58 completed in 2018 and an sNDA under FARXIGA NDA 202293 (s-018) was submitted as a rolling submission to the Division of Metabolism and Endocrinology Products (DMEP) on 14 November 2018 (eCTD sequence 0457), 27 November 2018 (eCTD sequence 0458), 29 November 2018 (eCTD sequence 0459), 12 December 2018 (eCTD sequence 0460) and 18 December 2018 (eCTD sequence 0461).

[b] HFpEF defined as LVEF >40%

[c] HFrEF defined as LVEF ≤40%

[d] CKD defined as eGFR ≥25 and ≤75 mL/min/1.7 m2 (CKD-EPI Formula)

[e] Albuminuria defined as increased albuminuria >3 months before visit 1 (enrollment) AND UACR ≥200 and ≤5000 mg/g at visit 1.

CKD chronic kidney disease. CV cardiovascular. FDA Food and Drug Administration. ID identification. IND investigational new drug. LVEF left ventricular ejection fraction. NCT national clinical trial. NDA new drug application. NYHA New York Heart Association. PDUFA prescription drug user fee act of 1992. T2DM type 2 diabetes mellitus. CKD chronic kidney disease. eGFR estimated glomerular filtration rate. UACR urine albumin creatinine ratio.

2 OBJECTIVES

The purpose of this patient-level meta-analysis is to provide additional data regarding the efficacy and safety of dapagliflozin as a treatment for patients with heart failure. Because the studies considered were powered for the primary endpoint, this meta-analysis allows evaluation of the components of a composite endpoint and important secondary efficacy endpoints that generally require more power than the individual trials can provide. This meta-analysis can provide a more precise estimate of the overall treatment effects in these efficacy outcomes and will allow evaluation of the impact of various intrinsic factors on the efficacy outcomes. This analysis also has the potential to support additional labelling claims.

This document describes one of the meta-analyses planned for the dapagliflozin clinical development program, in heart failure patients. Due to dapagliflozin's mechanism of action and the intersection across cardiovascular disease, diabetes and chronic kidney disease, it is important to have an integrated view of the potential beneficial effect of dapagliflozin across all outcome trials, thus additional meta-analyses are planned (eg, in CKD patients).

Objective	Variable
Primary Objective:	
To determine whether dapagliflozin is superior to placebo, when added to standard of care, in reducing the incidence of CV death	Time from randomization to the occurrence of deaths adjudicated as CV cause. Deaths adjudicated as 'cause undetermined' will be counted as CV deaths.
Secondary Objectives	
To determine whether dapagliflozin is superior to placebo, when added to standard of care, in reducing the incidence of all-cause mortality [a]	Time from randomization to death from any cause
To determine whether dapagliflozin is superior to placebo, when added to standard of care, on hospitalizations due to heart failure	Number of first and recurrent adjudicated HF hospitalizations, not including urgent HF visit<br><br>Time from randomization to the first occurrence of adjudicated HF hospitalization.
To determine if dapagliflozin compared with placebo reduces the incidence of a composite endpoint of CV death, myocardial infarction (MI), and ischemic stroke (or MACE)	Time to first occurrence of any of the components of this composite:<br>1 CV death<br>2 MI: fatal or non-fatal<br>3 Ischemic stroke: fatal or non-fatal
To evaluate the safety of dapagliflozin	The need for meta-analysis of safety variables will be based on evaluation of the individual studies.

[a] Mortality will be assessed as both an efficacy and a safety endpoint

[b] New onset of T2DM, post randomisation, is defined according to the following criteria: (1) Reporting of new onset of T2DM necessitating initiation of anti-diabetic medication, OR (2) HbA1c >6.5% (48 mmol/mol) measured by central lab at two consecutive study visits.

CV cardiovascular. eGFR estimated glomerular filtration rate. ESRD end stage renal disease. HbA1c glycated hemoglobin. HF heart failure. MI myocardial infarction. T2DM type 2 diabetes mellitus.

3 INTEGRATION OF EFFICACY AND SAFETY DATA

Data from the dapagliflozin clinical program will be integrated for all analyses. The following two pooling strategies will be conducted:

1. Data from the two HF outcome studies (D1699C00001 [DAPA-HF] and D169CC00001 [DELIVER]) will be combined in the integrated database and will be considered the Primary Pooling Set. These two studies are of similar design, have similar objectives, and incorporate mostly the same endpoints and frequency of assessments in patients with HFrEF and HFpEF, respectively.
2. The Secondary Pooling Set (Table 2) will be based on data from the studies selected based on the following inclusion criteria of studies. This Pooling Set captures a larger pool of data from HF patients compared to the Primary Pooling Set. Note: the studies listed in Table 2 are not exhaustive and there are other Phase 2 or 3 studies. However, the other studies did not meet all the inclusion criteria listed below and are, therefore, not included in this meta-analysis.

The Primary Pooling Set will be the primary source of data for all analyses. All analyses will be repeated using the Secondary Pooling Set as a sensitivity analysis. If results from the two pooling sets are different, for the same analysis (ie, one shows superiority and the other does not), the data will be further examined.

The analysis population is defined in Section 5.2.

Inclusion criteria:

- Studies that include heart failure patients.
- Studies that include heart failure endpoints.
- Studies that are Phase 3, randomized, double-blind with a controlled treatment period over 1 year to reflect an adequate treatment period of assessment.
- Studies that includes dapagliflozin 10 mg treatment arm.
- Studies with placebo as comparator.
- Background standard of care treatment.
- Dedicated CV or renal outcomes studies.
- Endpoints were collected as part of the design of the individual studies and generally the same frequency of assessments for efficacy and safety.

Table 2 Secondary Pooling Set

Criteria	DAPA-HF	DELIVER	DECLARE-TIMI 58	DETERMINE	DAPA-CKD
Studies that include heart failure patients.<br>(% of patients with HF at baseline).	X<br>(100%)	X<br>100%	X<br>(12%[a])	X<br>(100%)	X<br>(~11%[b])
Studies that include heart failure endpoints.	X	X	X	X	X
Phase 3, randomized, double-blind with a controlled treatment period over 1 year to reflect an adequate treatment period of assessment.	X	X	X		X
Includes dapagliflozin 10 mg treatment arm and placebo as comparator.	X	X	X	X	X
Background standard of care treatment.	X	X	X	X	X
Dedicated CV or renal outcomes studies	X	X	X		X
Endpoints were collected as part of the design of the individual studies and generally the same frequency of assessments for efficacy and safety.	X	X	X		X
**Overall**	**include**	**include**	**include**		**include**

a DECLARE-TIMI 58: Of the 17160 patients randomized: 1987 (12%) patients had HF [671 (3.9%) of patients had HFrEF (left ventricular ejection fraction [LVEF] ≤40%) and 1316 (7.7%) of patients had HF without known reduced EF (ie, were considered HFpEF; LVEF >40%).

b DAPA-CKD: the study is still ongoing, so exact numbers are unknown and the value of 11% is only approximate.

4 EFFICACY AND SAFETY VARIABLES

4.1 Endpoints to be included in the integrated analyses

The intent of the integrated analysis of the dapagliflozin clinical studies is to compare and to provide a more precise estimate of the treatment effect of dapagliflozin over placebo in patients with heart failure, as well as to provide a reliable assessment of consistency of treatment effect across subgroups of interest. The endpoints and outcome measures are listed in Table 1, with the hierarchy for testing as follows:

- Primary endpoint:
  - CV death
- Secondary endpoints:
  - All-cause mortality
  - Recurrent HF hospitalization
  - MACE

In all 4 outcome studies[1], events (such as CV death and hospitalization due to HF) are adjudicated by an independent Clinical Event Adjudication (CEA) committee. All adjudicated events from randomization until withdrawal of consent (WoC), or primary analysis censoring date (PACD), will be included in the analysis of these endpoints, except for the analysis of all-cause mortality, which also includes non-adjudicated deaths after WoC, but on or before PACD.

Events such as MI and ischemic stroke are also adjudicated by a CEA committee in DECLARE-TIMI 58, DAPA-HF, and DAPA-CKD, but not in the DELIVER study. Investigator reported cases of MI and ischemic stroke from DELIVER and the adjudicated cases from the other 3 studies will be used for the integrated analysis in both Primary Pooling and Secondary Pooling sets, and the composite endpoint MACE will be defined in a consistent way for the purpose of the integrated analysis.

For an additional description of the definition and derivation of these endpoints, refer to Section 3 of each individual study Statistical Analysis Plan (SAP).

[1] DECLARE-TIMI 58, DAPA-HF, DELIVER and DAPA-CKD

4.2 Definition of Subgroups

To explore the implications for efficacy among different patient subpopulations, the subgroups planned to be used in the integrated efficacy analysis, but are not limited to, are presented in Table 3.

Table 3 Characteristics and categories for subgroup analysis of the efficacy endpoints

Characteristic	Categories
Age (years)	≤ 65, >65
Sex	Male, female
Race	White, Black or African, Asian, other
NYHA class at enrollment [a]	II, III/IV
LVEF (%) at enrollment [b]	≤ 40, > 40
Type 2 diabetes mellitus at baseline [c]	Yes, No
Baseline eGFR (mL/min/1.73m^2)	<60, ≥60

[a] NYHA class not collected in DAPA-CKD. Subgroup analysis will only be conducted in the Primary Pooling Set
[b] LVEF not collected in DAPA-CKD. Subgroup analysis will only be conducted in the Primary Pooling Set
[c] Defined as history of T2DM or HbA1c ≥6.5% at both visit 1 and visit 2 for DAPA-HF, DECLARE and DAPA-CKD. Defined as T2DM at enrolment in DELIVER. The subgroup analysis by T2DM status will exclude T2DM as a stratification factor from the model.
eGFR estimated glomerular filtration rate. HbA1c glycated hemoglobin. LVEF left ventricular ejection fraction. NYHA New York Heart Association. T2DM type 2 diabetes mellitus.

4.3 Safety Evaluations

The safety and tolerability of dapagliflozin will be evaluated at the individual study level. The need for meta-analysis of any safety data will be assessed based the individual study evaluations.

5 ANALYSIS STRATEGY

5.1 General Principles

In general, summary tables of time-to-event analyses will include the number and percent of patients with event per treatment group, event rate, hazard ratio with 95% confidence interval and p-value. The event rate will be derived as the number of patients with event divided by the total duration of follow-up across all patients in a given group, presented as patients with event per 100 patient years.

Kaplan-Meier (KM) estimates of the cumulative proportion of patients with events will be calculated and plotted per treatment group, with the number of patients at risk indicated below the plot at specific time points. The KM plots will be presented for all time to event analyses.

Continuous data will be summarized using descriptive statistics: n, mean, standard deviation (SD), median, Q1, Q3, and range (ie, minimum and maximum), as appropriate.

For discrete, or ordinal data, percentages will be presented in a frequency table format. Summaries for some categorical variables will include 95% CIs for the difference in percent from placebo.

For additional description, refer to Section 4 of the individual study SAPs.

5.2 Analysis Population

Consistent with the definition in the individual study SAPs, all patients who have been randomized to study treatment will be included in the Full analysis set (FAS) irrespective of their protocol adherence and continued participation in the study. Patients will be analyzed according to their randomized IP assignment, irrespective of the treatment actually received. The FAS will be considered the primary analysis set for the efficacy variables.

All patients who received at least 1 dose of randomized treatment will be included in the safety population. Patients will be analyzed according to the treatment actually received. The Safety analysis set will be considered the primary analysis set for all safety variables.

5.3 Subject Characterization

5.3.1 Subject Identification

Each subject in the integrated analysis will be uniquely identified by the combination of study code, study site number, and subject number.

5.3.2 Subject Disposition

A disposition table of subjects will include the number and percentage of subjects in the full analysis set. Within the full analysis set, the number and percentage of subjects who completed and discontinued from studies will be summarized by treatment group.

5.3.3 Demographic and Baseline Characteristics

Integrated demographic and baseline characteristics data, including medical history, will be summarized, using frequency distributions and summary statistics based on the FAS, for each treatment group as well as for all patients combined. This will be done for both Pooling Set. No statistical test will be performed for comparison of any baseline measurement among treatment groups.

5.4 Efficacy Analyses

5.4.1 Hypotheses and Confirmatory Testing Procedure

For the time-to-events efficacy endpoints, the following null hypothesis will be tested:

H_0: Hazard Ratio [dapagliflozin:placebo] = 1

versus the alternative hypothesis

H_1: Hazard Ratio [dapagliflozin:placebo] ≠ 1

For the recurrent event endpoint, the following null hypothesis will be tested:

H_0: Rate Ratio [dapagliflozin:placebo] = 1

versus the alternative hypothesis

H_1: Rate Ratio [dapagliflozin:placebo] ≠ 1

To strongly control the familywise error rate at the 5% alpha level, a fixed sequence procedure including a pre-specified hierarchical order of the endpoints will be utilized. The testing procedure will continue down the hierarchy if the preceding endpoint is rejected at the 5% alpha level.

The following will be the order of the test:

1 CV death
2 All-cause mortality
3 Recurrent HF hospitalization
4 MACE

The time to first HF hospitalization will be a supportive analysis for the recurrent HF hospitalization endpoint and will not be included in the testing hierarchy.

5.4.2 Time-to-Event Endpoints

The primary analysis for the time to event endpoints such as HF hospitalization, CV death, all-cause mortality, and MACE will compare dapagliflozin versus placebo using a Cox proportional hazards model. The model will be stratified by study code and by T2DM status at randomization and will include a factor for treatment group. In addition, the endpoints of HF hospitalization and CV death will be adjusted for history of hospitalization for heart failure. Censoring rules from DAPA-HF (which are consistent with DELIVER) will be applied for all endpoints: HF hospitalization, CV death, MACE, and all-cause mortality across all studies.

The estimated treatment effect (ie, hazard ratio of dapagliflozin versus placebo), corresponding 95% confidence interval, and two-sided p-value for the HR will be presented. A sensitivity analysis will be performed with ejection fraction (≤40, >40) as a stratification factor instead of study code.

In order to investigate if the pooled studies are homogeneous, Cochrane's Q statistic, and Higgins and Thompsons' I^2 will be performed (Higgins et al 2003). Heterogeneity will be considered as follows:

- low if $I^2 \leq 25\%$
- moderate if $25\% < I^2 \leq 75\%$,
- high if $I^2 > 75\%$

Regardless of whether the heterogeneity is significant or not, the results from the individual studies will also be assessed for qualitative consistency to ensure meta-analysis proceeds in a scientific manner and to aid in the interpretation of the results from the meta-analysis.

The assumption of proportional hazards for the factor for treatment groups will be assessed visually using log-cumulative hazard plots. The effect of any departures from proportional hazards will be discussed as part of the presentation of results of the analyses and additional analysis may be performed to account for non-proportionality.

To explore the uniformity of the detected overall treatment effect on these outcome variables, subgroup analyses including testing for interaction between treatment and covariates will be performed in the subgroups. For each of the subgroup factors, a separate Cox proportional hazards model will be fitted using the same model terms as used for the primary analysis, with additional terms for the subgroup main effect and the treatment by subgroup interaction. Results will be presented in tabular form and forest plots.

5.4.3 Recurrent HF hospitalizations

For the analysis of total HF hospitalizations, occurrence of CV death can be regarded as semi-competing risk (informative censoring) and may introduce a bias in the treatment effect estimate for HF hospitalizations (dilution of effect size if the drug has a positive effect on both components). In order to address this concern and to account for the correlation between the two components, the joint modelling (frailty model) approach (Rogers et al 2016) will be used.

For joint frailty analysis the following assumptions are made (Liu et al 2004):

- As noted above, HF hospitalization and death cannot happen at the same day (only the CV death will be counted) and CV death is a terminating event, which prevents the occurrence of new HF hospitalizations.
- The hazard function of CV death (the instantaneous risk of death given that the subject is still alive) follows a proportional hazards model with constant baseline hazard, stratified by T2DM status at randomization and by study code, treatment and history of hospitalization for heart failure as covariates.
- The hazard function of recurrent HF hospitalizations (the instantaneous risk of having another HF hospitalization given that the subject is still alive) follows a proportional hazards model as well, with constant baseline hazard, stratified by T2DM status at randomization and by study code, treatment and history of hospitalization for heart failure as covariates.
- Within subject recurrent HF hospitalizations and CV death are correlated events. This correlation varies from subject to subject. To account for this correlation, the hazard function of recurrent HF hospitalizations and hazard function of CV death are modelled by a joint a subject specific random frailty term which follows a gamma distribution with unit mean and unknown variance.
- Censoring (including non-CV death) is noninformative, in particular it does not depend on random frailty.

Estimation in the frailty proportional hazards model will be done using a Gaussian quadrature approach (Liu and Huang 2008, Lu and Liu 2014, SAS Institure Inc. 2018 2018). Since SAS PROC NLMIXED supports only normal distributions, a probability integral transformation to generate numbers from a gamma distribution will be used (Nelson et al 2006) following (Liu and Huang 2008).

Additionally, non-parametric estimates of the marginal mean of the cumulative number of recurrent HF hospitalization rates over time will be calculated allowing for death as terminal event, and the estimates will be plotted (Ghosh and Lin 2000). The estimation will be done in SAS using the PROC PHREG and by treating the situation as competing risks with several records per subject (Andersen et al 2019).

5.4.4 Proportion of patients with a new diagnosis of T2DM in non-diabetic subpopulation at baseline

The proportion of patients with a new diagnosis of T2DM from a non-diabetic subpopulation at baseline will be analyzed by a logistic regression with study code, treatment group and baseline HbA1c (glycated hemoglobin). The odds ratio between treatment groups and its 95% confidence interval and corresponding two-sided p-value will be presented.

6 POWER CALCULATION

The number of events for each of the endpoints in this meta-analysis will be equivalent to the total number of events accrued from each of the outcome studies, which were individually powered for the evaluation of their primary endpoint(s). Figure 1 illustrates 80% or 90% power for testing based on the number of events that could be observed according to different assumed hazard ratios using a 2-sided alpha of 5% (dashed lines) and 1% (solid lines).

For example, if the assumed true HR is 0.80:

- For 800 total events observed, the study will have about 72% power (not shown) to detect an effect with 1% alpha and close to 90% power with 5% alpha
- For 1100 total events observed, the study will have close to 90% power to detect an effect with 1% alpha and about 96% power (not shown) with 5% alpha

Figure 1 Number of events to fall below upper bound of 1.0

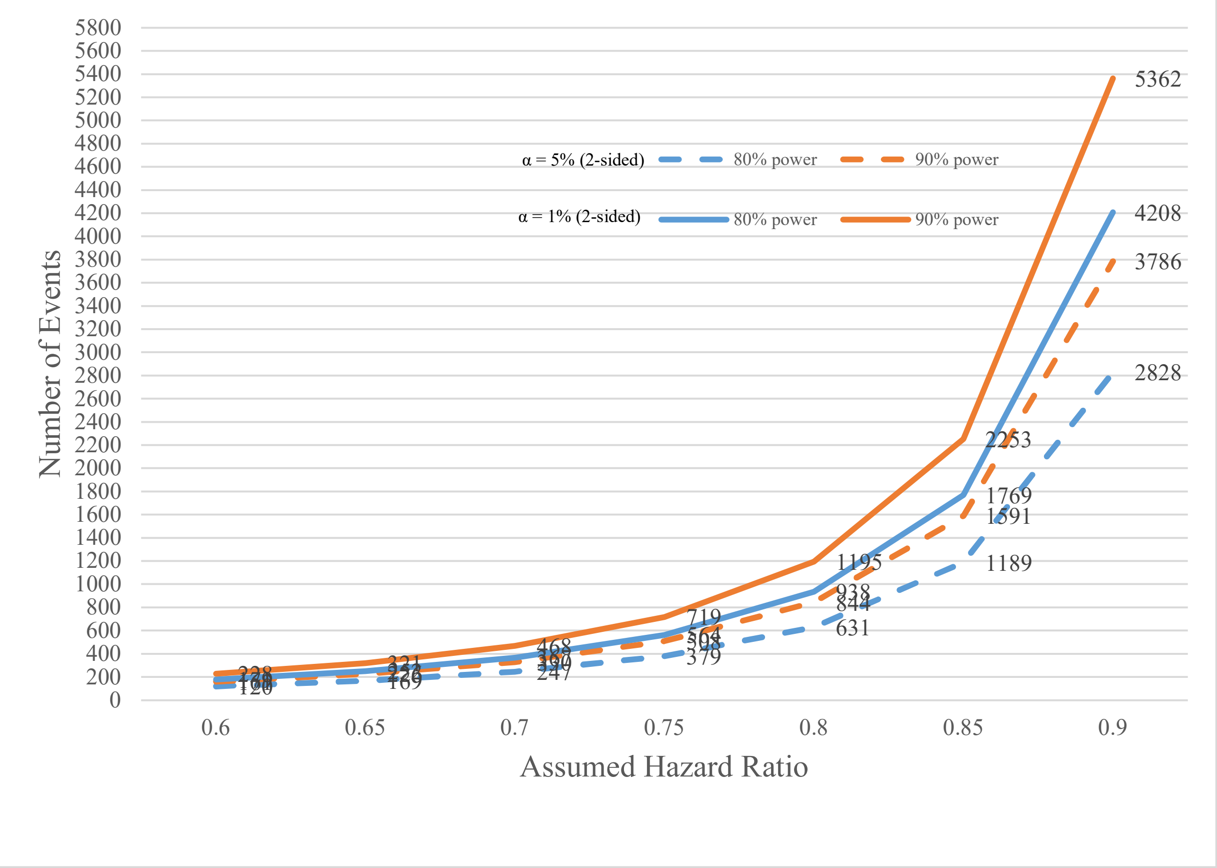

7 REFERENCES

Andersen PK, Angst J, Ravn H. Modeling marginal features in studies of recurrent events in the presence of a terminal event. Lifetime Data Anal. 2019.

Ghosh D, Lin DY. Nonparametric analysis of recurrent events and death. Biometrics. 2000;56(2):554-62.

Higgins JP, Thompson SG, Deeks JJ, Altman DG. Measuring inconsistency in meta-analyses. BMJ. 2003;327(7414):557-60.

Lam CSP, Voors AA, de Boer RA, Solomon SD, van Veldhuisen DJ. Heart failure with preserved ejection fraction: from mechanisms to therapies. Eur Heart J. 2018;39(30):2780-92.

Liu L, Huang X. The use of Gaussian quadrature for estimation in frailty proportional hazards models. Stat Med. 2008;27(14):2665-83.

Liu L, Wolfe RA, Huang X. Shared frailty models for recurrent events and a terminal event. Biometrics. 2004;60(3):747-56.

Lu L, Liu C. Analysis of Correlated Recurrent and Terminal Events Data in SAS. Proceedings of the Northeast SAS Users Group Conference 2008. 2014.

Nelson KP, Lipsitz SR, Fitzmaurice GM, Ibrahim J, Parzen M, Strawderman R. Use of the probability integral transformation to fit nonlinear mixed-effects models with nonnormal random effects. J Comp Graph Stat. 2006;15:39-57.

Rogers JK, Yaroshinsky A, Pocock SJ, Stokar D, Pogoda J. Analysis of recurrent events with an associated informative dropout time: Application of the joint frailty model. Stat Med. 2016;35(13):2195-205.

SAS Institure Inc. 2018. SAS/STAT 15.1 User's Guide. Cary, NC:SAS Institute Inc. 2018. Example 86.5:Failure Time and Frailty Model, page 7097. 2018.

Academic Statistical Analysis Plan

Study Code	D169CC00001
Edition Number	1.3
Date	April 15, 2022

DELIVER: Academic Statistical Analysis Plan

Dapagliflozin Evaluation to Improve the LIVEs of Patients with PReserved Ejection Fraction Heart Failure

An International, Double-blind, Randomised, Placebo-Controlled Phase III Study to Evaluate the Effect of Dapagliflozin on Reducing CV Death or Worsening Heart Failure in Patients with Heart Failure with Preserved Ejection Fraction (HFpEF)

DELIVER: Academic Statistical Analysis Plan

Dapagliflozin Evaluation to Improve the LIVEs of Patients with PReserved Ejection Fraction Heart Failure

An International, Double-blind, Randomised, Placebo-Controlled Phase III Study to Evaluate the Effect of Dapagliflozin on Reducing CV Death or Worsening Heart Failure in Patients with Heart Failure with Preserved Ejection Fraction (HFpEF)

DELIVER CO-CHAIRS, STEERING COMMITTEE

Scott D. Solomon, MD
Brigham and Women's Hospital

Scott Solomon (Apr 15, 2022 15:38 EDT) Apr 15, 2022

Signature Date

John J.V. McMurray, MD
University of Glasgow

Apr 15, 2022

Signature Date

Table of Contents

1. INTRODUCTION 4
2. CLINICAL ENDPOINTS OF INTEREST 4
3. LABORATORY-BASED ENDPOINTS OF INTEREST 5
4. BREAKDOWN OF ENDPOINTS 6
5. SUBGROUPS 6
5. ALTERNATIVE ANALYTIC APPROACHES 7
6. COVID-19 7
7. META-ANALYSES 9

1. INTRODUCTION

DELIVER is an international, multicentre, parallel group, event-driven, randomized, double-blind trial in patients with chronic heart failure and left ventricular ejection fraction (LVEF) >40%, comparing the effect of dapagliflozin 10 mg once daily, vs. placebo, in addition to standard of care. Patients with or without diabetes, with signs and symptoms of heart failure, a LVEF >40%, elevation in natriuretic peptides and evidence of structural heart disease are eligible. The primary endpoint is time-to-first cardiovascular death or worsening heart failure event (heart failure hospitalization or urgent heart failure visit), and will be assessed in dual primary analyses – the full population and in those with LVEF <60%. The study is event-driven and will target 1117 primary events. A total of 6,263 patients have been randomized.

The DELIVER executive committee has developed this academic statistical analysis plan (ASAP) that describes pre-specified analyses that were not described in the DELIVER regulatory SAP (rSAP). General principles outlined in the regulatory SAP will be followed unless specified otherwise here. This document is meant to supplement and complement the regulatory SAP and delineate all analyses that were pre-specified prior to database lock. When relevant, analyses will be conducted based on the pooled DAPA-HF and DELIVER dataset to examine the effects of dapagliflozin in a broad range of patients with HF.

2. CLINICAL ENDPOINTS OF INTEREST

In addition to the efficacy and safety variables listed in the rSAP, the effect of dapagliflozin on the following endpoints will be explored. These events that are imbalanced between arms may be analyzed as time-to-event to better understand the time course. All endpoints will be assessed in the full cohort and in the LVEF < 60% subgroup. These include:

- Days alive and out of the hospital
- Quality of life-adjusted days alive and out of the hospital
- Investigator reported vs. CEC-adjudicated endpoints
- Time to onset of benefit of dapagliflozin
- New diuretic initiation, discontinuation, and dose changes
- New onset atrial fibrillation
- In the T2D subgroup, new glucose lowering therapy initiation and changes in insulin dose (in those on insulin at baseline)
- In the non-T2D subgroup, new diagnosis of diabetes
- Signs and symptoms of HF
- Patient Global Impression of Severity
- Target risk factor control (for blood pressure, smoking, antiplatelet/anticoagulant therapy)
- Cardiac ischemic events including myocardial infarction, unstable angina, unplanned coronary revascularization, and stroke
- Hyperkalemia as a reported adverse event and initiation of new potassium-lowering therapy
- Acute kidney injury as a reported adverse event and initiation of dialysis
- Anemia and requirement for blood transfusion as reported adverse events
- Gout as an adverse event and initiation of new uric acid-lowering therapy
- KCCQ Overall Summary Score at 1, 4 and 8 months

- KCCQ Clinical Summary Score at 1, 4 and 8 months
- KCCQ Physical Limitations Score at 1, 4 and 8 months
- KCCQ Social Limitations Score at 1, 4 and 8 months
- Proportion of patients with clinically meaningful deterioration (5 point or greater worsening), and small (≥5 point), moderate (≥10 point) and large (≥20 point) improvement in KCCQ-TSS, CSS, OSS, PL, QoL and Social Limitations Scores.

<u>COVID-19 Related Endpoints</u>

In addition, the following COVID-19 related endpoints will be evaluated:

- Occurrence of COVID-19 infection (documented as AE or SAE)
- Occurrence of COVID-19 related hospitalizations (overall and among patients with COVID-19 infection)
- Occurrence of COVID-19 related hospitalizations requiring ICU admission (overall and among patients with Covid-19 infection)
- Occurrence of COVID-19 related deaths (overall and among patients with Covid-19 infection)
- Acute kidney injury and initiation of dialysis reported as an adverse event during hospitalization for COVID-19
- Requirement for mechanical ventilation reported as an adverse event during COVID-19 hospitalization
- Requirement for vasopressor support reported as an adverse event during COVID-19 hospitalization
- Sudden cardiac death/cardiac arrest requiring resuscitation during COVID-19 hospitalization
- Worsening heart failure reported during or following COVID-19 hospitalization
- Use of systemic corticosteroids for COVID-19
- Diabetic ketoacidosis reported as an adverse event during or following COVID-19 hospitalization
- Among patients with documented COVID-19 infection, total events of COVID-10 related hospitalizations and COVID-19 related deaths

3. LABORATORY-BASED ENDPOINTS OF INTEREST

In addition, the following laboratory-based endpoints will be assessed:

- eGFR-based
  - Composite of confirmed sustained decline in eGFR, ESRD, and/or renal death. Sustained decline in eGFR will be defined as ≥40%, ≥50%, ≥57% decline from baseline
  - Acute, chronic, and total eGFR slope analysis, including with blanking period to account for acute, expected eGFR changes
  - Focused examination of the “eGFR dip”, the acute changes in eGFR in the days-to-weeks after randomization
  - Recalculation of eGFR based on variable calculators (including the 2009 CKD-EPI Equation and 2021 CKD-EPI Equation)

4. BREAKDOWN OF ENDPOINTS

- Mode of death including focused examination of sudden death (as a composite with ventricular arrhythmias reported as adverse events)
- Reasons for hospitalization (total all-cause hospitalization, non-CV hospitalization, HF-related hospitalization, and other CV hospitalizations)
- 30-day readmission (all-cause and HF-related)
- Breakdown of worsening HF events (including urgent visits / Emergency Department stays / oral loop diuretic escalation)

Unknown deaths will not be included as a component of CV deaths in the primary analysis as outlined in the rSAP. In a prespecified exploratory analysis, we will apply a probabilistic model (predetermined prior to database lock) to better distinguish unknown deaths as either CV or non-CV in etiology. This probabilistic model will be built based on known clinical factors that differentially predict adjudicated known cases of CV vs. non-CV deaths.

5. SUBGROUPS

In addition to the subgroups listed in the rSAP, the following subgroups of interest will be explored to examine event rates and for consistency of efficacy and safety of dapagliflozin. All subgroups will be identified based on randomization or pre-randomization data unless otherwise specified. For each subgroup, we will assess the treatment effect and interaction with treatment for the primary endpoint and each of the secondary endpoints, including components of the primary endpoint, measures of quality of life (KCCQ), NYHA class, and the renal composite endpoint. In addition, all subgroups will be assessed in the LVEF < 60% subgroup.

- Improved/recovered LVEF (those who had LVEF ≤40% at any time prior to randomization)
- LVEF subgroups in the rSAP are specified according the following cutpoints (≤ 49%, 50 to 59%, ≥60%). Additional LVEF subgroups to limit digit preference will be considered and treatment effects will be examined across LVEF as a continuous function. In addition, the two-way interaction between sex and LVEF will be examined.
- Age subgroups in the rSAP are specified according to the following cutpoints (median age). Specific evaluation of older age categories will be considered and treatment effects will be examined across age as a continuous function
- BMI subgroups in the rSAP are specified according to the following cutpoints (30kg/m^2). BMI categories will additionally be evaluated according to the full WHO classification and treatment effects will be examined across BMI as a continuous function
- Other anthropometric indices e.g., waist-to-height ratio using quantiles and recognized cutpoints
- eGFR subgroups in the rSAP are specified according to the following cutpoints (60mL/min/1.73m^2). eGFR categories will additionally be evaluated according the full KDIGO classification and treatment effects will be examined across eGFR as a continuous function
- Focused examination of Stage IV CKD (if eGFR was less than 30mL/min/1.73m^2 at randomization or at any post-randomization measurement)
- Further breakdown of glycemic categories into no diabetes, prediabetes, and T2D and examination of treatment effects across HbA1c as a continuous measure
- Time from prior HF hospitalization

- Time from index HF diagnosis
- Background HF therapies including focused examination of patients on various combinations of therapies (including the Heart Failure Collaboratory score) and on/off MRA and on/off ARNI at randomization
- In T2D subgroup, background anti-hyperglycemic therapies including focused examination of patients on various combinations of therapies
- Patients with COPD
- Patients with OSA
- Patients with history of coronary artery disease / prior MI
- Patients with metabolic syndrome (using standard definitions)
- Subgroups based on baseline use and dosing of diuretics
- Patients with multimorbidity and frailty
- Patients with baseline risk as determined by the MAGGIC and other risk scores
- Subgroups based on baseline evidence of congestion and congestion scores
- Regional subgroups based on socioeconomic differences based on the GINI coefficient
- Subgroups based on KCCQ-TSS and other KCCQ domains at baseline.

5. ALTERNATIVE ANALYTIC APPROACHES

Unless otherwise specified, these alternative approaches will be considered for the primary endpoint and each of the secondary endpoints, including components of the primary endpoint, measures of quality of life (KCCQ), NYHA class, and the renal composite endpoint.

- Win ratio using different clinically relevant hierarchies e.g., death, heart failure hospitalization, urgent heart failure visit requiring IV therapy, outpatient therapy for worsening HF, quality of life, and kidney endpoints
- Multi-state modeling of changes in transitional states (ranging from alive and well to death)
- Estimation of time to first statistically significant benefit
- Forecasting lifetime benefit of dapagliflozin if treatment effects were assumed to be maintained long-term
- Absolute risk reductions and NNT calculation overall and across key subgroups
- Cost effectiveness based on US perspective, European perspective, and Other Regions of the World perspective
- Assessment of DELIVER trial and label eligibility in the GWTG-HF registry and other "real-world" datasets
- "Real world" application of the DELIVER trial findings to the GWTG-HF registry and other datasets to estimate projected benefit if dapagliflozin was implemented in usual care

6. COVID-19 META-ANALYSES

- Together with the subset of patients in DELIVER with COVID-19, a meta-analysis will be performed using available phase 3/4 published trials of sodium–glucose cotransporter 2 inhibitor therapies in COVID-19 (*including but not limited to DARE-19*)
- Analyses evaluating outcomes after post-randomization COVID-19 diagnosis will be performed (for instance, increase in mortality or HF event risk after COVID-19 diagnosis)

**Systemic Search**	To ensure trials beyond DARE-19 and DELIVER were not missed, a systemic search via PubMed and EMBASE will be conducted of<br>• Randomized, placebo-controlled trials of SGLT2 inhibitors in COVID-19<br>• Published between March 1st, 2020 to August 1, 2022
**Rationale**	DARE-19 randomized non-critically ill patients with one or more cardiometabolic risk factors (including T2D, HTN, ASCVD, HF or CKD) hospitalized with COVID-19 to dapagliflozin versus placebo, with one of the primary outcomes being respiratory/ cardiovascular/ kidney organ failure or death from any cause. DELIVER randomized patients with HF and LVEF above 40% to dapagliflozin or placebo, and due to the time course of the trial had many patients experiencing COVID-19 related hospitalizations and deaths. Neither trial was adequately powered to assess the effects of dapagliflozin on all-cause mortality, and specific end-organ complications. This pre-specified meta-analysis will allow for greater power to evaluate the effects of dapagliflozin on a range of COVID-19 related clinical endpoints.
**Overall Aim**	Using study-level published data from DARE-19 and participant-level data from DELIVER, we aim to estimate the effect of SGLT2 inhibitors on all-cause mortality and specific end-organ complications overall, and in clinically-relevant subgroups
**Primary Endpoint**	COVID-19 related death (this includes COVID-19 related deaths in DELIVER and all deaths in DARE-19)
**Secondary Endpoints**	• Acute kidney injury and initiation of dialysis during or following hospitalization for COVID-19<br>• Requirement for mechanical ventilation during COVID-19 hospitalization<br>• Requirement for vasopressor support during COVID-19 hospitalization<br>• Sudden cardiac death/resuscitated cardiac arrest requiring resuscitation during COVID-19 hospitalization<br>• Worsening heart failure during or following COVID-19 hospitalization<br>• Composite of COVID-19 related death and organ failure (acute kidney injury, initiation of dialysis, mechanical ventilation, vasopressor support, cardiac death/ resuscitated cardiac arrest, worsening heart failure)<br>• Composite of COVID-19 related death, acute kidney injury and initiation of dialysis.<br>• Diabetic ketoacidosis during or following COVID-19 hospitalization
**Subgroups**	• With or without diabetes<br>• With or without ASCVD<br>• With or without CKD (eGFR < 60)<br>• With or without HTN<br>• Age, sex, race, BMI, geographic region
**Statistical Analysis**	• Intention-to-treat analyses from both trials will be considered and include all randomized participants<br>• All effect sizes will be extracted as point estimates (95% CI).<br>• Statistical heterogeneity will be assessed between trials
**Risk of Bias**	Study quality will be evaluated using the Cochrane Risk of Bias Tool
**Reporting**	This planned meta-analysis will be conducted and reported in accordance with the Preferred Reporting Items for Systematic Reviews and Meta-Analysis statement
**Registration**	This meta-analysis will be registered on PROSPERO

7. META-ANALYSIS OF SGLT2 INHIBITOR HFPEF TRIALS AND OTHER SGLT2 INHIBITOR TRIALS

A meta-analysis will be performed using available phase 3/4 published trials of other sodium–glucose cotransporter 2 inhibitor therapies in HFpEF, including but not limited to EMPEROR-Preserved.

**Systemic Search**	To ensure trials beyond EMPEROR-Preserved and DELIVER were not missed, a systemic search via PubMed and EMBASE will be conducted of<br>• Randomized, placebo-controlled CV and kidney outcomes trials of SGLT2 inhibitors<br>• Published between January 1, 2015 to July 1, 2022<br>• Only studies including >1,000 patients with HF and LVEF >40%
**Rationale**	Both EMPEROR-Preserved and DELIVER were similarly designed in evaluating patients with HF, an LVEF above 40%, and elevated natriuretic peptides. Neither trial was powered for mortality or kidney disease outcomes. This pre-specified meta-analysis of the 2 largest trials of HFmrEF and HFpEF will allow for greater power to evaluate a broad range of clinical endpoints and within subgroups of interest than either trial could provide alone.
**Overall Aim**	Using study-level published data from EMPEROR-Preserved and participant-level data from DELIVER, we aim to estimate the effect of SGLT2 inhibitors on cardiovascular events, kidney events, and mortality outcomes overall, and in clinically-relevant subgroups
**Primary Endpoint**	Time from randomization to the occurrence of the composite of death adjudicated as CV cause or unplanned HF hospitalization
**Secondary Endpoints**	• Time from randomization to the occurrence of the composite of death adjudicated as CV cause or a worsening HF event (including either unplanned hospitalization or urgent HF visit requiring IV therapy)<br>• Total number of worsening HF events and cardiovascular death<br>• Time from randomization to the occurrence of deaths adjudicated as CV cause<br>• Time from randomization to death from any cause<br>• Time from randomization to renal composite outcome (50% or higher sustained decline in eGFR, end stage kidney disease, or renal death)<br>• Proportion of patients with clinically meaningful deterioration (5 point or greater worsening), and small (≥5 point), moderate (≥10 point), and large (≥15 point) improvement in KCCQ-TSS, CSS, OSS
**Subgroups**	• LVEF (<50%, ≥50 to <60%, ≥60%)<br>• With or without diabetes<br>• Use of no use of ACEi/ARB/ARNI at baseline<br>• Use and no use of MRA at baseline<br>• Age (≥70 and <70 years), sex (male, female), race (White, Black, Asian, Other), BMI (<30 and ≥30 kg/m^2), eGFR (≥60 and <60mL/min/1.73m^2), systolic blood pressure, history of AF/AFL, hospitalization for HF within 12 months, NYHA class (II and III/IV)
**Statistical Analysis**	• Fixed effects model<br>• Only intention-to-treat analyses from both trials will be considered and include all randomized participants<br>• All effect sizes will be extracted as point estimates (95% CI). For the time-to-first event endpoints, Cox proportional hazards models will be used for hazard ratio (HR) and 95% CI. Recurrent event analyses will be based on the Lin-Wei-Yang-Ying model and summarized as

	rate ratio (RR) and 95% CI. Responder analyses for KCCQ changes will be based on logistic regression analyses summarized as odds ratios with 95% CIs.<br>• The continuous association between LVEF and treatment effects on the primary endpoint will be assessed with restricted cubic spline analyses. Data from these published splines in the EMPEROR program will be digitized using a validated, semiautomatic tool (DigitizeIt software https://www.digitizeit.xyz/).<br>• Statistical heterogeneity will be assessed between trials
**Risk of Bias**	Study quality will be evaluated using the Cochrane Risk of Bias Tool
**Reporting**	This planned meta-analysis will be conducted and reported in accordance with the Preferred Reporting Items for Systematic Reviews and Meta-Analysis statement
**Registration**	This meta-analysis will be registered on PROSPERO

Meta-analyses will also be performed using available phase 3/4 published trials of other sodium–glucose cotransporter 2 inhibitor therapies in different disease states to provide a comprehensive assessment of the value of SGLT2 inhibitors across the disease spectrum.
