# Supplementary material for: Dapagliflozin across the range of ejection fraction in patients with heart failure: a patient-level, pooled meta-analysis of DAPA-HF and DELIVER
Source: Nat Med. 2022 Aug 27;28(9):1956–64. doi: 10.1038/s41591-022-01971-4 (PMC9499855; doi:10.1038/s41591-022-01971-4)
Supplement: Supplementary file 2 — Reporting Summary [file 41591_2022_1971_MOESM2_ESM.pdf]

nature research

Corresponding author(s): John McMurray

Last updated by author(s): Jul 26, 2022

# Reporting Summary

Nature Research wishes to improve the reproducibility of the work that we publish. This form provides structure for consistency and transparency in reporting. For further information on Nature Research policies, see our Editorial Policies and the Editorial Policy Checklist.

## Statistics

For all statistical analyses, confirm that the following items are present in the figure legend, table legend, main text, or Methods section.

| n/a | Confirmed | |
|---|---|---|
| ☐ | ☒ | The exact sample size (*n*) for each experimental group/condition, given as a discrete number and unit of measurement |
| ☐ | ☒ | A statement on whether measurements were taken from distinct samples or whether the same sample was measured repeatedly |
| ☐ | ☒ | The statistical test(s) used AND whether they are one- or two-sided<br>*Only common tests should be described solely by name; describe more complex techniques in the Methods section.* |
| ☐ | ☒ | A description of all covariates tested |
| ☐ | ☒ | A description of any assumptions or corrections, such as tests of normality and adjustment for multiple comparisons |
| ☐ | ☒ | A full description of the statistical parameters including central tendency (e.g. means) or other basic estimates (e.g. regression coefficient) AND variation (e.g. standard deviation) or associated estimates of uncertainty (e.g. confidence intervals) |
| ☐ | ☒ | For null hypothesis testing, the test statistic (e.g. *F*, *t*, *r*) with confidence intervals, effect sizes, degrees of freedom and *P* value noted<br>*Give P values as exact values whenever suitable.* |
| ☒ | ☐ | For Bayesian analysis, information on the choice of priors and Markov chain Monte Carlo settings |
| ☒ | ☐ | For hierarchical and complex designs, identification of the appropriate level for tests and full reporting of outcomes |
| ☒ | ☐ | Estimates of effect sizes (e.g. Cohen's *d*, Pearson's *r*), indicating how they were calculated |

*Our web collection on statistics for biologists contains articles on many of the points above.*

## Software and code

Policy information about availability of computer code

| | |
|---|---|
| Data collection | No software used |
| Data analysis | Stata version 17.0 (College Station, TX, USA) and SAS version 9.4 (Cary, USA) |

For manuscripts utilizing custom algorithms or software that are central to the research but not yet described in published literature, software must be made available to editors and reviewers. We strongly encourage code deposition in a community repository (e.g. GitHub). See the Nature Research guidelines for submitting code & software for further information.

## Data

Policy information about availability of data

All manuscripts must include a data availability statement. This statement should provide the following information, where applicable:

- Accession codes, unique identifiers, or web links for publicly available datasets
- A list of figures that have associated raw data
- A description of any restrictions on data availability

Data underlying the findings described in this manuscript may be obtained following AstraZeneca's data sharing policy described at https://astrazenecagrouptrials.pharmacm.com/ST/Submission/Disclosure.

# Field-specific reporting

Please select the one below that is the best fit for your research. If you are not sure, read the appropriate sections before making your selection.

☒ Life sciences ☐ Behavioural & social sciences ☐ Ecological, evolutionary & environmental sciences

For a reference copy of the document with all sections, see nature.com/documents/nr-reporting-summary-flat.pdf

# Life sciences study design

All studies must disclose on these points even when the disclosure is negative.

**Sample size**

Sample size was 11007, 4744 from DAPA-HF and 6263 from DELIVER. The sample size and power calculations are described in the following:

1: Solomon SD, de Boer RA, DeMets D, Hernandez AF, Inzucchi SE, Kosiborod MN, Lam CSP, Martinez F, Shah SJ, Lindholm D, Wilderäng U, Öhrn F, Claggett B, Langkilde AM, Petersson M, McMurray JJV. Dapagliflozin in heart failure with preserved and mildly reduced ejection fraction: rationale and design of the DELIVER trial. Eur J Heart Fail. 2021 Jul;23(7):1217-1225. doi: 10.1002/ejhf.2249.

2: McMurray JJV, Solomon SD, Inzucchi SE, Køber L, Kosiborod MN, Martinez FA, Ponikowski P, Sabatine MS, Anand IS, Bělohlávek J, Böhm M, Chiang CE, Chopra VK, de Boer RA, Desai AS, Diez M, Drozdz J, Dukát A, Ge J, Howlett JG, Katova T, Kitakaze M, Ljungman CEA, Merkely B, Nicolau JC, O'Meara E, Petrie MC, Vinh PN, Schou M, Tereshchenko S, Verma S, Held C, DeMets DL, Docherty KF, Jhund PS, Bengtsson O, Sjöstrand M, Langkilde AM; DAPA-HF Trial Committees and Investigators. Dapagliflozin in Patients with Heart Failure and Reduced Ejection Fraction. N Engl J Med. 2019 Nov 21;381(21):1995-2008. doi: 10.1056/NEJMoa1911303.

**Data exclusions**

None.

**Replication**

Prospectively designed clinical trials.

**Randomization**

Both were double blind placebo controlled prospective randomized trials.

**Blinding**

Both trials were double blind.

# Reporting for specific materials, systems and methods

We require information from authors about some types of materials, experimental systems and methods used in many studies. Here, indicate whether each material, system or method listed is relevant to your study. If you are not sure if a list item applies to your research, read the appropriate section before selecting a response.

## Materials & experimental systems

| n/a | Involved in the study | |
|---|---|---|
| ☒ | ☐ | Antibodies |
| ☒ | ☐ | Eukaryotic cell lines |
| ☒ | ☐ | Palaeontology and archaeology |
| ☒ | ☐ | Animals and other organisms |
| ☐ | ☒ | Human research participants |
| ☐ | ☒ | Clinical data |
| ☒ | ☐ | Dual use research of concern |

## Methods

| n/a | Involved in the study | |
|---|---|---|
| ☒ | ☐ | ChIP-seq |
| ☒ | ☐ | Flow cytometry |
| ☒ | ☐ | MRI-based neuroimaging |

## Human research participants

Policy information about studies involving human research participants

**Population characteristics**

Each enrolled patients with a diagnosis of heart failure, functional limitation, and elevated natriuretic peptides. The principal difference between the two trials was that patients with an ejection fraction of 40% or less were randomized in DAPA-HF and those with an ejection fraction greater than 40% in DELIVER. In both trials, patients were randomized to dapagliflozin at a dose of 10 mg once daily, or a matching placebo, in addition to standard care. The full characteristics of each trial have been described in detail in the following publications:

1: Solomon SD, de Boer RA, DeMets D, Hernandez AF, Inzucchi SE, Kosiborod MN, Lam CSP, Martinez F, Shah SJ, Lindholm D, Wilderäng U, Öhrn F, Claggett B, Langkilde AM, Petersson M, McMurray JJV. Dapagliflozin in heart failure with preserved and mildly reduced ejection fraction: rationale and design of the DELIVER trial. Eur J Heart Fail. 2021 Jul;23(7):1217-1225. doi: 10.1002/ejhf.2249.

2: McMurray JJV, Solomon SD, Inzucchi SE, Køber L, Kosiborod MN, Martinez FA, Ponikowski P, Sabatine MS, Anand IS, Bělohlávek J, Böhm M, Chiang CE, Chopra VK, de Boer RA, Desai AS, Diez M, Drozdz J, Dukát A, Ge J, Howlett JG, Katova T,

| | |
|---|---|
| | Kitakaze M, Ljungman CEA, Merkely B, Nicolau JC, O'Meara E, Petrie MC, Vinh PN, Schou M, Tereshchenko S, Verma S, Held C, DeMets DL, Docherty KF, Jhund PS, Bengtsson O, Sjöstrand M, Langkilde AM; DAPA-HF Trial Committees and Investigators. Dapagliflozin in Patients with Heart Failure and Reduced Ejection Fraction. N Engl J Med. 2019 Nov 21;381(21):1995-2008. doi: 10.1056/NEJMoa1911303. |
| Recruitment | Patients in New York Heart Association (NYHA) functional class II to IV, with a left ventricular ejection fraction (LVEF) of 40% or less and an elevated N-terminal pro-B-type natriuretic peptide (NT-proBNP) level were eligible for DAPA-HF. Participants were also required to receive guideline-recommended treatments for heart failure with reduced ejection fraction. The main exclusions to enrolment were a history of type 1 diabetes mellitus, hypotension causing symptoms or a systolic blood pressure less than 95 mmHg, and an estimated glomerular filtration rate (eGFR) less than 30 mL/min/1.73m2. |
| Ethics oversight | The Ethics Committee of the participating institutions approved the protocols, and all patients gave written informed consent. |

Note that full information on the approval of the study protocol must also be provided in the manuscript.

# Clinical data

Policy information about clinical studies

All manuscripts should comply with the ICMJE guidelines for publication of clinical research and a completed CONSORT checklist must be included with all submissions.

| | |
|---|---|
| Clinical trial registration | NCT03036124 and NCT03619213 |
| Study protocol | Statistical plans have been submitted with manuscript. See also,<br><br>1: Solomon SD, de Boer RA, DeMets D, Hernandez AF, Inzucchi SE, Kosiborod MN, Lam CSP, Martinez F, Shah SJ, Lindholm D, Wilderäng U, Öhrn F, Claggett B, Langkilde AM, Petersson M, McMurray JJV. Dapagliflozin in heart failure with preserved and mildly reduced ejection fraction: rationale and design of the DELIVER trial. Eur J Heart Fail. 2021 Jul;23(7):1217-1225. doi: 10.1002/ejhf.2249.<br><br>2: McMurray JJV, Solomon SD, Inzucchi SE, Køber L, Kosiborod MN, Martinez FA, Ponikowski P, Sabatine MS, Anand IS, Bělohlávek J, Böhm M, Chiang CE, Chopra VK, de Boer RA, Desai AS, Diez M, Drozdz J, Dukát A, Ge J, Howlett JG, Katova T, Kitakaze M, Ljungman CEA, Merkely B, Nicolau JC, O'Meara E, Petrie MC, Vinh PN, Schou M, Tereshchenko S, Verma S, Held C, DeMets DL, Docherty KF, Jhund PS, Bengtsson O, Sjöstrand M, Langkilde AM; DAPA-HF Trial Committees and Investigators. Dapagliflozin in Patients with Heart Failure and Reduced Ejection Fraction. N Engl J Med. 2019 Nov 21;381(21):1995-2008. doi: 10.1056/NEJMoa1911303. |
| Data collection | Data were collected at study sites by investigators and by AstraZeneca during each trial, but were analyzed independently at the University of Glasgow. The DAPA-HF trial randomized patients between 5 February 2017and 17 August 2018, with patients enrolled in at 410 sites in 20 countries. Enrollment in DELIVER began on 27 August 2018 and the last patient was randomized on 18 January 2021, with patients enrolled at 353 sites, in 20 countries . See also<br>1: Solomon SD, de Boer RA, DeMets D, Hernandez AF, Inzucchi SE, Kosiborod MN, Lam CSP, Martinez F, Shah SJ, Lindholm D, Wilderäng U, Öhrn F, Claggett B, Langkilde AM, Petersson M, McMurray JJV. Dapagliflozin in heart failure with preserved and mildly reduced ejection fraction: rationale and design of the DELIVER trial. Eur J Heart Fail. 2021 Jul;23(7):1217-1225. doi: 10.1002/ejhf.2249.<br>2. McMurray JJV, DeMets DL, Inzucchi SE, Køber L, Kosiborod MN, Langkilde AM, Martinez FA, Bengtsson O, Ponikowski P, Sabatine MS, Sjöstrand M, Solomon SD; DAPA-HF Committees and Investigators. The Dapagliflozin And Prevention of Adverse-outcomes in Heart Failure (DAPA-HF) trial: baseline characteristics. Eur J Heart Fail. 2019 Nov;21(11):1402-1411. doi: 10.1002/ejhf.1548. Epub 2019 Jul 15. |
| Outcomes | Both trials were event-driven and had the same primary endpoint which was a composite of the time to the first occurrence of worsening heart failure or death from a cardiovascular cause. Worsening heart failure was defined as unplanned hospital admission for heart failure or an urgent visit for worsening heart failure resulting in the administration of an intravenous diuretic.<br>In the original "regulatory" statistical analysis plan for the meta-analysis (dated 2 August 2019), a pre-specified hierarchy of endpoints was provided with control of alpha (see statistical analysis below). The endpoints were: death from cardiovascular causes; death from any cause; total (i.e., first and repeat) hospital admissions for heart failure (with an additional supportive analysis of time to the first occurrence of hospital admissions for heart failure, outside alpha control); and the composite of death from cardiovascular causes, myocardial infarction, or stroke ("major adverse cardiovascular events" [MACE]). Because of the possible attenuation of the benefit of SGLT2 inhibition at higher ejection fractions reported in the EMPEROR trials8 (as described in the introduction), we also examined the composite outcome used in the EMPEROR trials i.e., time to the first occurrence of hospital admission for worsening heart failure or death from cardiovascular causes in our analyses. In both trials an independent Cardiovascular Endpoint Committee (CEC), blinded to treatment allocation, adjudicated all deaths and non-fatal cardiovascular events submitted by investigators (or otherwise identified) as possible endpoints using a charter reflecting the 2017 Cardiovascular and Stroke Endpoint Definitions for Clinical Trials developed by the Standardized Data Collection for Cardiovascular Trials Initiative<br><br>1.Hicks KA, Mahaffey KW, Mehran R, Nissen SE, Wiviott SD, Dunn B, Solomon SD, Marler JR, Teerlink JR, Farb A, Morrow DA, Targum SL, Sila CA, Hai MT, Jaff MR, Joffe HV, Cutlip DE, Desai AS, Lewis EF, Gibson CM, Landray MJ, Lincoff AM, White CJ, Brooks SS, Rosenfield K, Domanski MJ, Lansky AJ, McMurray JJ, Tcheng JE, Steinhubl SR, Burton P, Mauri L, O'Connor CM, Pfeffer MA, Hung HM, Stockbridge NL, Chaitman BR, Temple RJ; Standardized Data Collection for Cardiovascular Trials Initiative (SCTI) . 2017 Cardiovascular and stroke endpoint definitions for clinical trials. Circulation 2018;137:961–972<br>Please see statistical plans submitted. |
